# Differential Response of Gestational Tissues to TLR3 Viral Priming Prior to Exposure to Bacterial TLR2 and TLR2/6 Agonists

**DOI:** 10.3389/fimmu.2020.01899

**Published:** 2020-08-25

**Authors:** Zahirrah B. M. Rasheed, Yun S. Lee, Sung H. Kim, Ranjit K. Rai, Camino S. M. Ruano, Eberechi Anucha, Mark H. F. Sullivan, David A. MacIntyre, Phillip R. Bennett, Lynne Sykes

**Affiliations:** ^1^Imperial College Parturition Research Group, Department of Metabolism, Digestion and Reproduction, Imperial College London, London, United Kingdom; ^2^Department of Obstetrics and Gynaecology, Faculty of Medicine, University of Malaya, Kuala Lumpur, Malaysia; ^3^March of Dimes European Preterm Birth Research Centre, Imperial College London, London, United Kingdom; ^4^INSERM U1016 Institut Cochin, Paris, France

**Keywords:** toll like receptor, viral priming, preterm labour, pregnancy, inflammation

## Abstract

**Background:** Infection/inflammation is an important causal factor in spontaneous preterm birth (sPTB). Most mechanistic studies have concentrated on the role of bacteria, with limited focus on the role of viruses in sPTB. Murine studies support a potential multi-pathogen aetiology in which a double or sequential hit of both viral and bacterial pathogens leads to a higher risk preterm labour. This study aimed to determine the effect of viral priming on bacterial induced inflammation in human *in vitro* models of ascending and haematogenous infection.

**Methods:** Vaginal epithelial cells, and primary amnion epithelial cells and myocytes were used to represent cell targets of ascending infection while interactions between peripheral blood mononuclear cells (PBMCs) and placental explants were used to model systemic infection. To model the effect of viral priming upon the subsequent response to bacterial stimuli, each cell type was stimulated first with a TLR3 viral agonist, and then with either a TLR2 or TLR2/6 agonist, and responses compared to those of each agonist alone. Immunoblotting was used to detect cellular NF-κB, AP-1, and IRF-3 activation. Cellular TLR3, TLR2, and TLR6 mRNA was quantified by RT-qPCR. Immunoassays were used to measure supernatant cytokine, chemokine and PGE2 concentrations.

**Results:** TLR3 (“viral”) priming prior to TLR2/6 agonist (“bacterial”) exposure augmented the pro-inflammatory, pro-labour response in VECs, AECs, myocytes and PBMCs when compared to the effects of agonists alone. In contrast, enhanced anti-inflammatory cytokine production (IL-10) was observed in placental explants. Culturing placental explants in conditioned media derived from PBMCs primed with a TLR3 agonist enhanced TLR2/6 agonist stimulated production of IL-6 and IL-8, suggesting a differential response by the placenta to systemic inflammation compared to direct infection as a result of haematogenous spread. TLR3 agonism generally caused increased mRNA expression of TLR3 and TLR2 but not TLR6.

**Conclusion:** This study provides human *in vitro* evidence that viral infection may increase the susceptibility of women to bacterial-induced sPTB. Improved understanding of interactions between viral and bacterial components of the maternal microbiome and host immune response may offer new therapeutic options, such as antivirals for the prevention of PTB.

## Introduction

Preterm birth (PTB) occurs in 5–18% of pregnancies worldwide, with rates varying depending on geography, ethnicity, and lifestyle factors (1). PTB causes approximately 1 million neonatal deaths per year (1), and is the leading cause of mortality in children under the age of five (2). The associated neonatal morbidity leads to a global social and financial burden, with the estimated annual cost of PTB being $26 billion in the USA alone in 2007 (3). While many risk factors for PTB have been identified, the underlying aetiology and biological mechanisms are poorly understood. The lack of progress in the development of new therapeutic strategies to prevent PTB is partly due to the multiple aetiological factors that drive it. Between 30–35% of PTBs are medically indicated. The remaining PTBs result from spontaneous preterm labour (sPTL) and/or preterm premature rupture of membranes (PPROM) (4). Of the women who experience PPROM or sPTL, it is widely accepted that both systemic and local infection/inflammation are major causal factors, especially in early PTB (5–7).

The maternal fetal interface may come into contact with pathogens ascending from the lower reproductive tract or haematogenously spread as part of a systemic illness. Maternal illnesses such as pyelonephritis (8), appendicitis (9), and pneumonia (10), are associated with spontaneous preterm labour (sPTL), and many animal models have demonstrated that systemic delivery of pathogens will induce PTL (11, 12). In women, systemic infection by *Listeria monocytogenes* has been reported to cause sPTL in approximately a third of infected women (13). Hematogenous spread of organisms to the placenta has been postulated to explain the association between periodontal disease and PTL, with oral cavity microorganisms being isolated from amniotic fluid (AF) as a result of transplacental passage (14).

Using standard culture techniques, as few as 1% of women are found to have bacteria in their amniotic fluid at term prior to the onset of labour (15). However, microbial invasion of the amniotic cavity (MIAC) occurs in as many as 50% of women with an open cervix (16), and 32% of women with PPROM (17), placing them at significantly increased risk of preterm delivery (18, 19). The microbes commonly detected in these studies include *Ureaplasma urealyticum*, Group B *streptococcus, Mycoplasma hominis*, and *Gardnerella vaginalis*, and *Lactobacillus* species, which are all commonly found in the vagina. Several studies using bacterial DNA sequencing approaches have confirmed the importance of vaginal microbial composition in the risk of PPROM and PTL (20–23).

There is substantial evidence to support a role for inflammation of the fetal membranes, myometrium and cervix in the mechanisms of parturition, contributing to membrane rupture, uterine contractility and cervical ripening in both term and sPTL (24). Inflammation involves recruitment of leukocytes and the production of cytokines, chemokines, and prostaglandins. An augmented inflammatory response is seen in cases of PTL, with higher concentrations of Interleukin (IL)-6, IL-1β, and IL-8 in amnion, choriodecidua and in placenta compared to term labour (25). Furthermore, high concentrations of IL-6 in cervicovaginal fluid associates with an increased risk of preterm delivery (26). In microbial driven inflammation, these responses are initiated upon recognition of unique molecular structures found on the microorganisms by pattern recognition receptors (PRR) such as Toll-like receptors (TLRs). Once bound by their respective ligands, TLRs in the lower reproductive tract and placenta trigger downstream signalling cascades via the inflammatory transcription factors NF-κB and AP-1 (27). These transcription factors play a key role in regulating the expression of pro-labour (matrix metalloproteinases (MMPs), COX-2 and prostaglandins) and pro-inflammatory mediators (IL-1β, TNF-α, IL-8, and IL-6) (28–31). In turn, these mediators lead to cervical remodelling, membrane rupture, and uterine contractility.

Toll like receptors (TLRs) are transmembrane proteins with extracellular domains containing highly conserved leucine-rich repeat motifs. TLR1, 2, 4, 5, and 6 recognize bacterial pathogen associated molecular patterns (PAMPs), whereas TLR3, 7, 8, and 9 mediate viral recognition. Transcripts from all ten TLRs are detected in placental explants, decidua and amnion epithelial cells (AECs) (32–34), and various studies have reported on their expression in vaginal and cervical cells, as well as uterine smooth muscle cells, as reviewed by Nasu and Narahara (35). Support for the functional role of TLRs in microbial driven sPTL comes from their increased expression at the maternal-fetal interface in women with chorioamnionitis (36, 37), and the ability of TLR agonists to induce PTB in animal models (38, 39). Gram-positive bacterial products are the main ligands of the TLR2 receptor, including Group B *streptococcus* (*S. agalactiae*) and *Ureaplasma urealyticum*, which are both associated with sPTL (40). Intraperitoneal administration of Lipoteichoic acid (LTA) or Peptidoglycan (PGN) induces sPTL in the mouse (41). TLR4 recognizes lipopolysacharride (LPS) motifs found on most Gram-negative bacteria, such as *Escherichia coli* and *Mycoplasma hominis*, both of which are associated with sPTL. LPS is a potent inducer of sPTL in the mouse, an effect which is mitigated in TLR-4 mutant mice, supporting the key functional role of TLR4 (42).

In contrast to the causal link between bacterial infection and sPTL, the role of viruses is less well established. However, HIV, hepatitis B, and RSV infection during pregnancy are associated with an increased risk of PTB (43–45). Histological chorioamnionitis has been reported to be more common in preterm placentas positive for adenovirus compared to both adenovirus negative preterm placentas and adenovirus positive term placentas (46). However, the most compelling evidence for the role of viruses in inducing sPTB is the ability of viral TLR agonists to induce sPTB in animal models. A dose dependent increase in preterm delivery rates is seen in mice treated with the TLR3 agonist poly I:C, an effect which is mitigated in TLR3 knockout mice (47).

Mouse models have demonstrated a synergistic effect of bacterial and viral TLR agonists in induction of inflammation and sPTL (39, 48–50). These studies lend support to the concept of multi-pathogen induced preterm labour, and the possibility that viruses can increase susceptibility to bacterial induced preterm labour. To explore whether this concept might apply in humans, we tested the “double-hit” hypothesis in *in vitro* human cell models of ascending infection and haematogenous infection. Vaginal epithelial cells lines (VECs), primary amnion epithelial cells (AECs) and primary myocytes (ascending infection model), or peripheral blood mononuclear cells and placental explants (haematogenous model) were primed with the TLR3 agonist poly I:C prior to treatment with the TLR2 agonist heat-killed *Listeria Monocytogenes* (HKLM) or TLR2/6 agonist FSL-1 to determine their effect on pro-inflammatory and pro-labour mediators.

## Materials and Methods

### Ethics Statement

Placenta and myometrial biopsies were collected in accordance with Ethical Approval from Hammersmith, Queen Charlotte's & Chelsea Hospitals Research Ethics Committee (Ref 2002/628) and Riverside Research Ethics Committee (Ref 3358), respectively. Peripheral blood collection was approved by the South East London Ethics Committee (Ref 10/H0805/54). Informed written consent was obtained from all participants prior to obtaining the samples.

### Reagents

The agonists for TLR2 (heat-killed *Listeria monocytogenes*, HKLM, Cat tlr1-hklm), and TLR2/6 (FSL-1, Pam2CGDPKHPKSF, Cat tlr1-fsl) were purchased from Invivogen, (Toulouse, France), the TLR3 agonist polyinosinic-polycytidylic acid, Poly I:C, Cat P9582) was purchased from Sigma-Aldrich (Gillingham, UK). Total RNA was extracted using TRIzol™ (Invitrogen Life Technologies) or the RNeasy Micro Kit (Qiagen). All other reagents for cDNA synthesis and quantitative PCR were purchased from Sigma-Aldrich (Gillingham, UK). All primers were from Thermo-Scientific (Waltham, MA). Table 1 shows sequences used for amplification of target genes. Antibodies to detect the phosphorylated p-65 NF-κB subunit Cat #3031 (AB_330559), phosphorylated c-Jun subunit Cat #9164 (AB_330892), phosphorylated IRF3 Cat #29047 (AB_2773013), and ß-actin Cat #A5441 (AB_476744) were purchased from Cell Signalling Technology (Beverly, MA) and Sigma-Aldrich (Gillingham, UK), respectively. The Meso Scale Discovery Immunoassay was used to detect a panel of 9 cytokines and chemokines MSD (Rockville, MD). The Prostaglandin E2 parameter assay kit (KGE004B) was purchased from R&D Systems (Minneapolis, MN).

**Table 1:** Primers sequences for RT-QPCR.

**Genes**	**Forward primer**	**Reverse primer**
TLR2	TGCTGCCATTCTCATTCTTCTG	AGGTCTTGGTGTTCATTATCTTCC
TLR3	CCTGGTTTGTTAATTGGATTAACGA	TGAGGTGGAGTGTTGCAAAGG
TLR6	GCCACCATGCTGGTGTTGGCT	CGCCGAGTCTGGGTCCACTG
ß-actin	AGGCATCCTCACCCTGAAGTA	CACACGCAGCTCATTGTAGA

### *In-vitro* Cell and Tissue Explant Culture and Treatment Protocols

The vaginal epithelial cell line VK2/E6E7 was purchased from American Type Culture Collection (ATCC^®^ CRL2616™). All samples of placenta, myometrial biopsies, and peripheral blood were collected at the time of planned pre-labour caesarean section from healthy women at term. The indications for caesarean delivery were either previous caesarean section or breech presentation.

#### Preparation of Vaginal Epithelial Cells (VECs)

Cells from ATCC were stored in liquid nitrogen until the time of cell culture. Cells were thawed for 1 min at 37°C in a water bath and immediately transferred into pre-warmed Dulbecco's Modified Eagle's medium (DMEM) containing 10% fetal calf serum (FCS). The suspension was centrifuged at 125 × *g* for 5 min to pellet the cells and resuspended in keratinocyte serum free media (KSFM) supplemented with bovine pituitary extract (BPE), epidermal growth factor (EGF) and calcium chloride (CaCl_2_). Cells were grown in T25 culture flasks at 37°C, 5% CO_2_ until confluent prior to treatment with viral and bacterial agonists (Table 2). Cells were grown to a maximum of passage seven and all experiments were repeated a minimum of three times.

**Table 2:** Concentration and duration of TLR agonist treatments.

**Cell/Tissue type**	**TLR3 agonist Poly I:C**	**TLR2 agonist HKLM**	**TLR 2/6 agonist FSL-1**
VECs	25 μg/ml for 6 h	10^5^ cells/ml for 24 h	0.01 μg/ml for 24 h
AECs	25 μg/ml for 12 h	10^5^ cells/ml for 24 h	0.01 μg/ml for 24 h
Myocytes	25 μg/ml for 12 h	10^5^ cells/ml for 24 h	0.1 μg/ml for 24 h
PBMCs	5 μg/ml for 30 min	10^8^ cells/ml for 18 h	0.1 μg/ml for 18 h
Placental explants	25 μg/ml for 24 h	10^8^ cells/ml for 24 h	0.1 μg/ml for 24 h

#### Preparation of Amnion Epithelial Cells (AECs)

AECs were processed as described previously (51). Placenta was collected within 1 h of delivery and processed immediately. The amnion layer was separated from the choriodecidua, cut into strips and washed in phosphate buffer saline (PBS) then incubated in pre-warmed 0.5 mM of EDTA-PBS for 15 min at room temperature. The strips were then rinsed in PBS and further incubated for 50 min at 37°C in 60 mls of pre-warmed dispase (Gibco) (2 g/L in PBS) to digest the intracellular matrix. The strips were shaken vigorously in pre-warmed DMEM containing 10% FCS, 2 mM/L L-glutamine, 100 U/ml penicillin, and 100 μg/ml streptomycin for 4 min to isolate the AECs from the intracellular matrix. Tissue strips were then removed, and the solution was centrifuged at 2,000 rpm for 10 min at room temperature to pellet the cells. Pelleted cells were resuspended in pre-warmed medium and grown in plates at 5% CO_2_ until confluent and ready for TLR agonist treatment. Once cells reached 80% confluence, they were serum starved in DMEM containing 1% FCS, 2 M/L L-glutamine, 100 U/ml penicillin and 100 μg/ml in preparation for TLR agonist treatment. A minimum of three and maximum of six biological replicates were used for each experiment.

#### Preparation of Myocytes

Myometrial biopsies were taken from the upper margin of the lower segment incision and prepared as previously described (52). The biopsies were mechanically dissected and myocytes were isolated by incubating the dissected tissue in 10 mg of collagenase 1 A, 10 g collagenase X and 200 mg of bovine serum albumin (BSA) in 30 mls of 1:1 DMEM and F-12 HAM for 45 min at 37°C. DMEM containing 10% FCS, was added to inactivate the enzymes and the suspension was filtered through a 100 μm cell strainer then centrifuged at 3,000 rpm for 5 min. Pelleted cells were resuspended in (DMEM) containing 10% FCS, 2 mM/L L-glutamine, 100 U/ml penicillin and 100 μg/ml streptomycin and cultured in T25 culture flasks until confluent. Cells were used for experiments up until passage 6. Prior to TLR agonist treatment, cells were serum starved in DMEM containing 1% FCS, 2 mM/L L-glutamine, 100 U/ml penicillin and 100 μg/ml. Three to six biological replicates were used for each experiment.

#### Preparation of Peripheral Blood Mononuclear Cells (PBMCs)

PBMCs were isolated as described previously (53). Blood was processed within 30 mis of collection and was diluted 1:1 ratio with PBS and layered on top of Ficoll-Paque^TM^ PLUS (GE Healthcare, Uppsala, Sweden) and centrifuged at 400 × *g* for 40 min at room temperature. The cloudy halo formed in the middle containing PBMC was extracted and washed twice with PBS for 10 min at 400 × *g*. The pelleted cells were resuspended in complete RPMI 1640 culture medium (Invitrogen Life Technologies, Grand Island, NY) containing 10% FCS, 2 mM/L L-glutamine, 100 U/ml penicillin and 100 μg/ml of streptomycin and cultured in 24 well plates at a concentration of 1.7 × 10^5^ /ml.

#### Preparation of Placental Explants

Placentas were collected and processed immediately after delivery for explant studies and processed as previously described (54). Several 2 cm^3^ tissue samples were cut, washed in PBS to remove excess blood, and further dissected to samples of 20–30 mg weight. Samples were incubated in 24 well plates containing RPMI 1640 culture medium supplemented with 10% FCS, 2 mM/L L-glutamine, 100 U/ml Penicillin and 100 μg/ml Streptomycin in 8% oxygen and 5% CO_2._ Medium was changed every 48 h and explants were serum starved on day 4 prior to TLR agonist treatment on day 5 to allow for syncytiotrophoblast regeneration.

#### Treatment Protocols

- To examine the activation of NF-κB (p-p65), AP-1 (p-c-Jun) and IRF3 (p-IRF3) proteins, VECs, AECs, myocytes, PBMCs and placental explants were treated with poly I:C over a timecourse of 30 min, 1, 4, 6, 12, and 24 h.
- To determine whether TLR3 receptor priming led to an augmented pro-inflammatory and pro-labour response in cells then exposed to TLR2 or TLR2/6 agonist, cells were treated with either vehicle control or poly I:C (TLR3 agonist). Following a wash out, cells were then treated with either vehicle control, HKLM (TLR2 agonist) or FSL (TLR 2/6 agonist). Fold changes of cytokine, chemokine and prostaglandin E2 (PGE2) were compared to baseline concentrations, and comparisons between cells treated with vehicle alone, poly I:C alone, HKLM or FSL-1 alone, and priming with poly I:C prior to HKLM or FSL-1 were made.
- To determine the effect of poly I:C on TLR2, 3, and 6 mRNA expression level, each cell type and explant were treated with poly I:C for up to 24 h. mRNA expression was examined at 0, 30 min, 1 h, 4 h, 6 h, 12 h, and 24 h.

### Western Blot Analysis

NF-κB, AP-1, and IRF-3 activation were analysed in all cell types; VEC, AECs, myocytes, PBMCs and placental explants after poly I:C stimulation by western blotting. Cells and tissues were lysed with whole cell lysis buffer (Cell Signalling) containing 5 μl/mL of protease inhibitor cocktail (Sigma Aldich), 5 μl/mL of phosphatase inhibitor cocktail (Sigma Aldich) and 1 mM of phenylmethylsulfonyl fluoride (PMSF). Lysed cells were incubated on ice for 5 min and centrifuged for 10 min at 14,000 × *g* at 4°C. The supernatant was collected, and protein concentration was determined using Bradford method with BSA as the standard. SDS Page gel electrophoresis was run and the protein was transferred onto polyvinylidene difluoride (PVDF) membranes (300 mA, 1.5 h, 4°C) and blocked in 5% milk in 1 × tris-buffered saline with tween (TBS-T) for 1 h at room temperature. The PVDF membrane was incubated either with phosphorylated p65 rabbit polyclonal antibody (Cell Signalling, #3031) for NF-κB, phosphorylated c-Jun rabbit polyclonal antibody (Cell Signalling, #9164) for AP-1, phosphorylated IRF3 rabbit monoclonal antibody (Cell Signalling, #29047) or anti ß-actin mouse monoclonal antibody (Sigma Aldrich, A5441) overnight at 4°C. The blots were washed 3 times with 1 × TBS-T before incubated with diluted horseradish-peroxidase-conjugated secondary antibodies, 1:2,000 p-p65, p-c-Jun and p-IRF3, Cell Signalling, and 1:25,000 for ß-actin, Santa Cruz]) for 1 h at room temperature. Blots were then washed 3 times with 1x TBS-T and proteins were detected using Clarity ECL Western (BioRad) or Luminata Forte (Millipore) blotting substrate of horseradish peroxidase.

### mRNA Expression of Toll Like Receptors 2, 3, and 6

Total RNA was extracted from VECs, AECs, myocytes and placental explants, using TRIzol™ (Invitrogen, CA, USA), whereas PBMCs RNA was extracted using the RNeasy Micro Kit (Qiagen) according to the manufacturer's instructions. Total RNA was synthesised into cDNA as previously described (51). Relative quantification of *TLR2, 3*, and *6* gene expression was performed using real- time PCR performed on an Applied Biosystems StepOne Real-Time PCR system using SYBR^®^ Green Master Mix (Applied Biosystems, Foster City, CA). Primers sequences were examined with BLAST software against the National Center for Biotechnology Information database and are summarised in Table 1. Water non-template controls were used, and correctly sized amplified products were confirmed using gel electrophoresis. The comparative C_T_ method (ΔΔ*C*_T_) was used for relative quantification, and results were calculated relative to baseline gene expression and expression of the ß-actin housekeeping gene.

### Quantification of Cytokines, Chemokines and PGE2

Supernatant from cultured cells and explants was used to analyse IL-6, IL-8, IL-10, IL-1α, IL-1ß, TNF-α, IL-4, MIP-1α, MIP-1ß, and RANTES production. Supernatant was collected at the following timepoints; baseline, following TLR3 treatment, and following TLR2 or TLR2/6 treatment. Supernatant was also analysed from single agonist treatment and from vehicle control samples. Quantification of the cytokines and chemokines were performed using the Meso Scale Discovery platform. U-Plex kits were used according to manufacturer's instructions, MSD (Meso Scale Diagnostics, Rockville, Maryland). Plates were read by the QuickPlex SQ 120 (Meso Scale Diagnostics).

Supernatant was also used to analyse PGE2 at the same timepoints. The PGE2 ELISA kit was used according to the manufacturer's guidelines (KGE004B, R&D Systems). Briefly supernatant was diluted 1:3 with the assay diluent and plated alongside the calibration curve standards (Range of 39–2,500 pg/ml). The optical density of each well and readings from the absorbance value of 450 nm was subtracted from 540 and 570 nm in order to obtain PGE2 concentrations.

### Statistical Analysis

All statistical analyses were performed using GraphPad Prism 8 (GraphPad Software, San Diego, CA). The statistical tests were selected depending on the number of groups analysed and the distribution of the data. One-way ANOVA was used when comparing more than two groups of one variable if data was normally distributed, or by using the Kruskal-Wallis for non-parametric distributions. A Two-way ANOVA was used when comparing data with more than one variable for both data found to be normally distributed, and for experiments in which experimental replicates were <5. The *Bonferroni* or *Dunnett's post hoc* tests were used as appropriate. A *p* < 0.05 was considered to indicate statistical significance.

## Results

### TLR3 Induced Activation of NF-κB, AP-1, and IRF in VECs, AECs, and Myocytes

**Figure 1:**
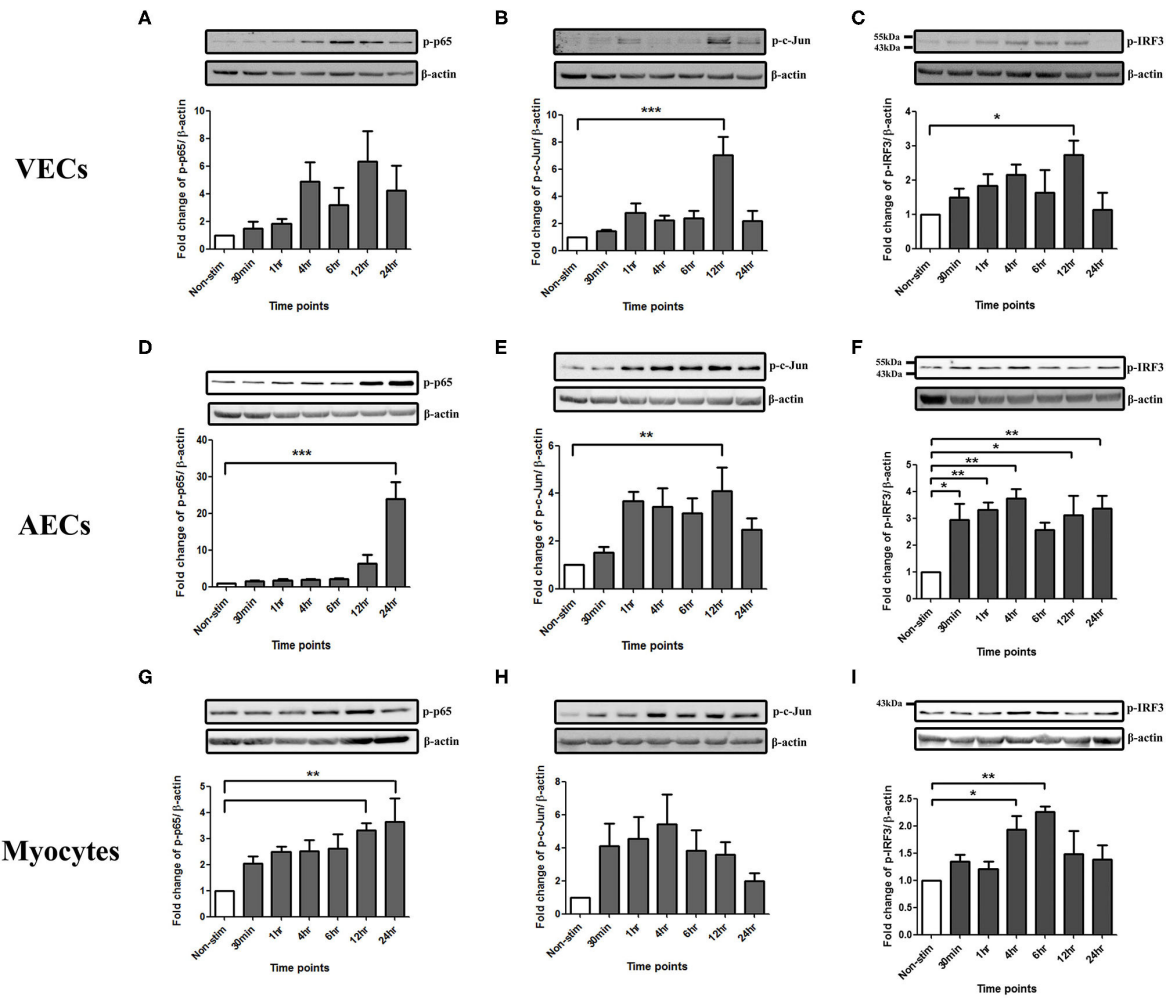
Activation of NF-κB, AP-1, and IRF in VECs, AECs, and myocytes. VECs, AECs, and myocytes were treated with poly I:C for 30 min, 1, 4, 6, 12, and 24 h and phospho-p-65, phospho-c-Jun and phospho-IRF3 were examined. In VECs, no statistical significance was observed with p-p65 expression **(A)**, however, p-c-Jun **(B)**, and p-IRF3 **(C)** increased significantly at 12 h (*p* < 0.001 and *p* < 0.05, respectively) with 25 μg/ml. An increase in p-p65 (*p* < 0.001) **(D)** and p-c-Jun (*p* < 0.01) **(E)** at 24 and 12 h, respectively, was observed in AECs. 25 μg/ml. An earlier significant increase of p-IRF3 activation also was seen at 30 min (*p* < 0.05), 1, 4 (*p* < 0.01), 12 (*p* < 0.05), and 24 h (*p* < 0.01) **(F)**. In myocytes, p-p65 increased significantly at 12 and 24 h (*p* < 0.01) **(G)** whereas a non-significant increase was observed in p-c-Jun at 30 min, 1, 2, 6, and 12 h **(H)** with 10 μg/ml. Significant increase was also observed in p-IRF3 at 4 (*p* < 0.05) and 6 h (*p* < 0.01) **(I)** with 25 μg/ml. For statistical analysis, One-way ANOVA was used for normally distributed data and Kruskal-Wallis was used for data not normally distributed with Dunnett's multiple comparison test *n* = 3–8. ^*^ = *p* < 0.05, ^**^ = *p* < 0.01, ^***^ = *p* < 0.001.

To study the effect of poly I:C on the activation of the transcription factors NF-κB, AP-1 and p-IRF3 in VECs, AECs and myocytes cells were stimulated with poly I:C for a total of 24 h, and cells harvested at appropriate intervals to determine phosphorylated transcription factor levels at 30 min, 1 h, 4 h, 6 h, 12 h, and 24 h. A significant increase in p-c-Jun and p-IRF-3 was seen in VECs by 12 h (*p* < 0.001 and *p* < 0.05, respectively) (Figures 1B,C), and a trend of increased p-p65 was seen, although this was not significant with 25 μg/ml (Figure 1A). All transcription factors were significantly activated in AECs with the earliest activation seen with p-IRF3 (*p* < 0.01) (Figures 1D–F) with 25 μg/ml of poly I:C. A significant increase in p-p65 by 12 h was seen myocytes treated with 10 μg/ml poly I:C, which was sustained until 24 h (*p* < 0.01) (Figure 1G), no significant change was seen in p-c-jun (Figure 1H), and early activation of p-IRF3 was seen at 4 h and 6 h with 25 μg/ml poly I:C (*p* < 0.05 and *p* < 0.01) (Figure 1I).

### Model of Ascending Infection: TLR3 Agonist Viral Priming Augments TLR2/6 Agonist Induced Pro-inflammatory and Pro-labour Mediators in VECs, AECs, and Myocytes

**Figure 2:**
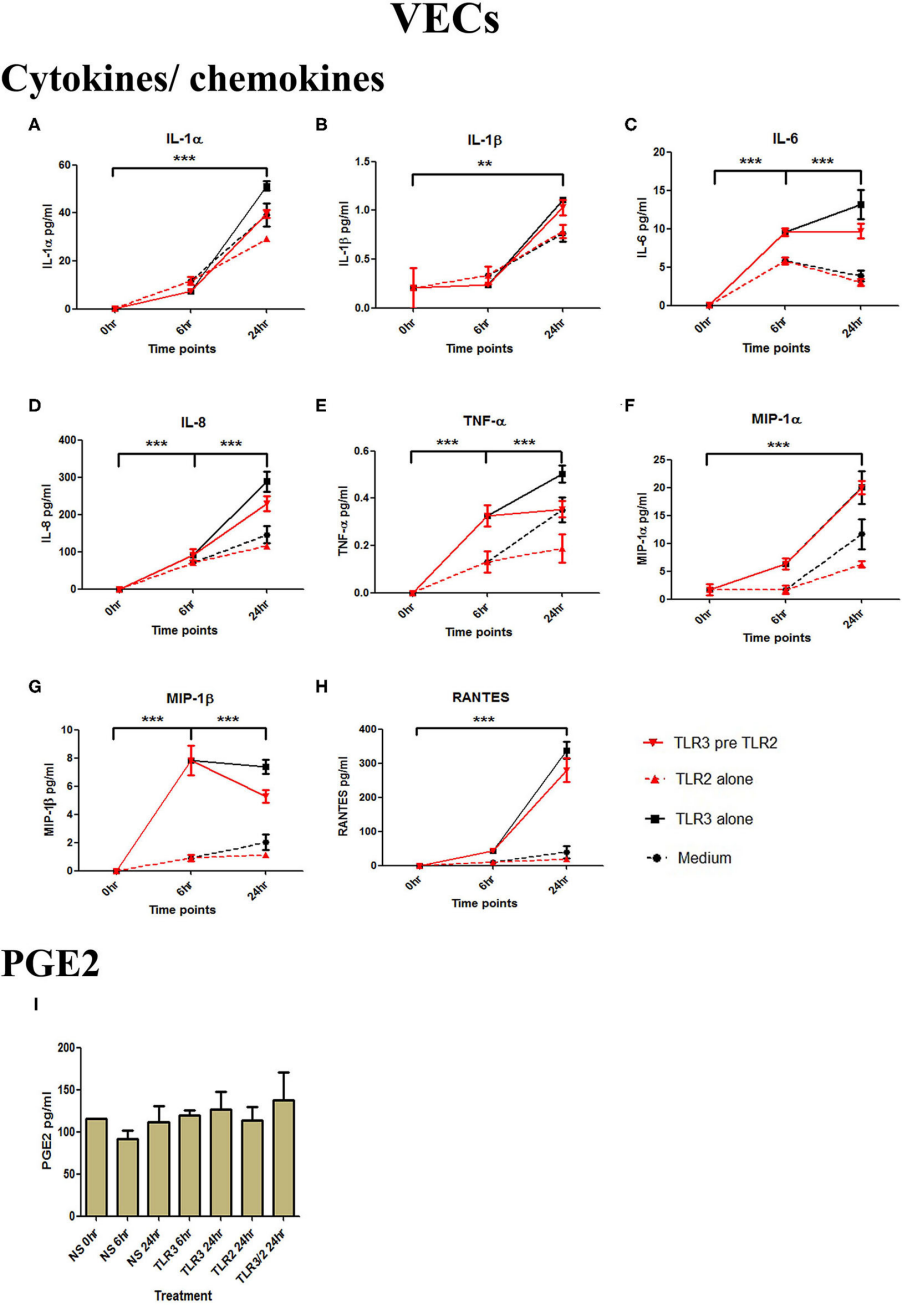
The effect of TLR3 viral priming on TLR2 agonist induced pro-inflammatory and pro-labour mediators in vaginal epithelial cells. Monolayer VECs were primed with 25 μg/ml of poly I:C for 6 h prior to 10^5^ cells/ml of HKLM for 24 h and the supernatant was collected to quantify the pro-inflammatory and pro-labour mediators. Poly I:C stimulation alone significantly increased the production of all cytokines and chemokines. TLR3 priming prior to HKLM stimulation showed no augmentation in IL-1α **(A)**, IL-1ß **(B)**, IL-6 **(C)**, IL-8 **(D)**, TNF-α **(E)**, MIP-1α **(F)**, MIP-1ß **(G)**, RANTES **(H)**, and PGE2 **(I)**. For statistical analysis Two-way ANOVA with Bonferroni's multiple comparison test was used. ^*^ = effect of TLR3 compared to 0 h. ^**^ = *p* < 0.01, ^***^ = *p* < 0.001.

In a model of ascending infection, VECs, AECs, and myocytes were treated with bacterial TLR agonists. Heat-killed *Listeria Monocytogenes* (HKLM) (10^5^ cells/ml for 1 h) was used as a pure TLR2 agonist, and FSL-1 (0.01 μg/ml in VECs and AECs for 1 h or 0.01 μg/ml in myocytes for 4 h) was used as a TLR2/TLR6 agonist (Supplementary Figure 1, Figure 2). HKLM led to a significant increase in both phospho-p65 and p-c-Jun in all cell types. FSL-1 led to a significant increase in phospho-p65 and p-c-Jun in VECs and AECs, but only p-c-Jun in myocytes.

#### TLR3 Priming Prior to TLR2 Agonist Treatment in Model of Ascending Infection

**Figure 3:**
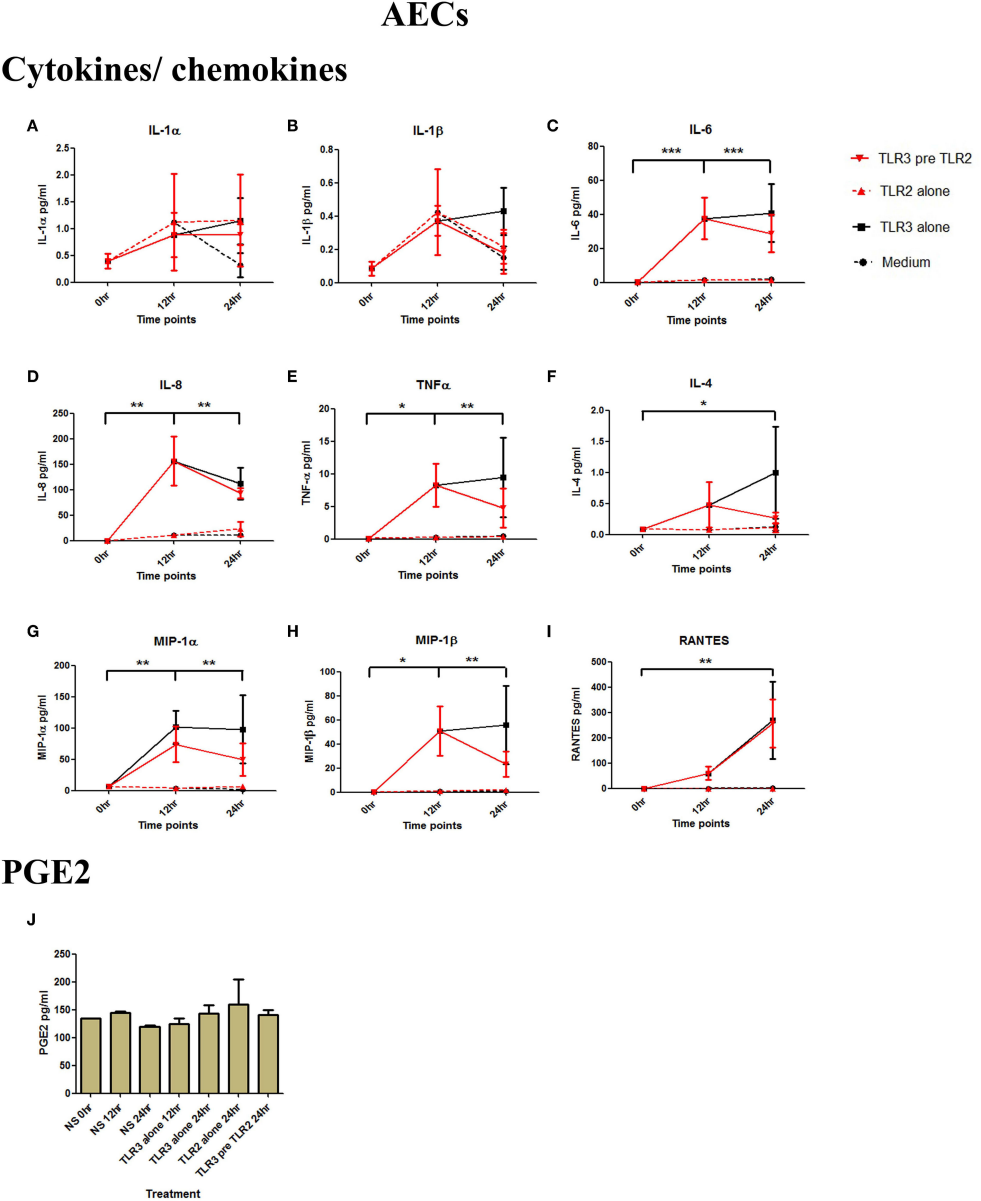
The effect of TLR3 viral priming on TLR2 agonist induced pro-inflammatory and pro-labour mediators in amnion epithelial cells. Monolayer cells were primed with 25 μg/ml of poly I:C for 12 h prior to 10^5^ cells/ml of HKLM for 24 h. Supernatant was collected to quantify the pro-inflammatory and pro-labour mediators. Poly I:C stimulation alone significantly increased the production of IL-6, IL-8, TNF-α, IL-4, MIP-1α, MIP-1ß, and RANTES. TLR3 priming prior to HKLM stimulation showed no augmentation in IL-1α **(A)**, IL-1ß **(B)**, IL-6 **(C)**, IL-8 **(D)**, TNF-α **(E)**, IL-4 **(F)**, MIP-1α **(G)**, MIP-1ß **(H)**, RANTES **(I)**, and PGE2 **(J)**. For statistical analysis Two-way ANOVA with Bonferroni's multiple comparison test was used. ^*^ = effect of TLR3 compared to 0 h. For statistical analysis Two-way ANOVA with Bonferroni's multiple comparison test was used. ^*^ = effect of TLR3 compared to 0 h. ^*^ = *p* < 0.05, ^**^ = *p* < 0.01, ^***^ = *p* < 0.001.

VECs were treated with 25 μg/ml of poly I:C or vehicle for 6 h prior to treatment with 10^5^ cells/ml of HKLM or vehicle. No significant changes in IL-1α or IL-1β were seen with any treatment condition (Figures 3A,B). Treatment with the TLR3 agonist alone significantly increased the production of pro-inflammatory cytokines IL-1α (*p* < 0.001), IL-1β (*p* < 0.01), IL-6, IL-8, TNF-α (*p* < 0.001), and the chemokines MIP-1α, MIP-1β, and RANTES (*p* < 0.001) (Figures 2A–H). HKLM treatment alone or with poly I:C priming did not change cytokine or chemokine concentrations. Similarly, there was no increase in PGE2 production in VECs following any of the treatment conditions (Figure 2I).

AECs were treated with 25 μg/ml of poly I:C or vehicle for 12 h prior to treatment with 10^5^ cells/ml of HKLM or vehicle. Poly I:C led to a significant increase in IL-6 (*p* < 0.001), IL-8 (*p* < 0.01), TNF-α, MIP-1α, MIP-1β, and RANTES (*p* < 0.01), and IL-4 (*p* < 0.05) (Figures 3C–I). As with VECs, there was no increase following incubation with 10^5^ cells/ml of HKLM or following sequential incubation with poly I:C then by HKLM. Furthermore, no increased production of PGE2 was seen with any treatment combination (Figure 3J).

**Figure 4:**
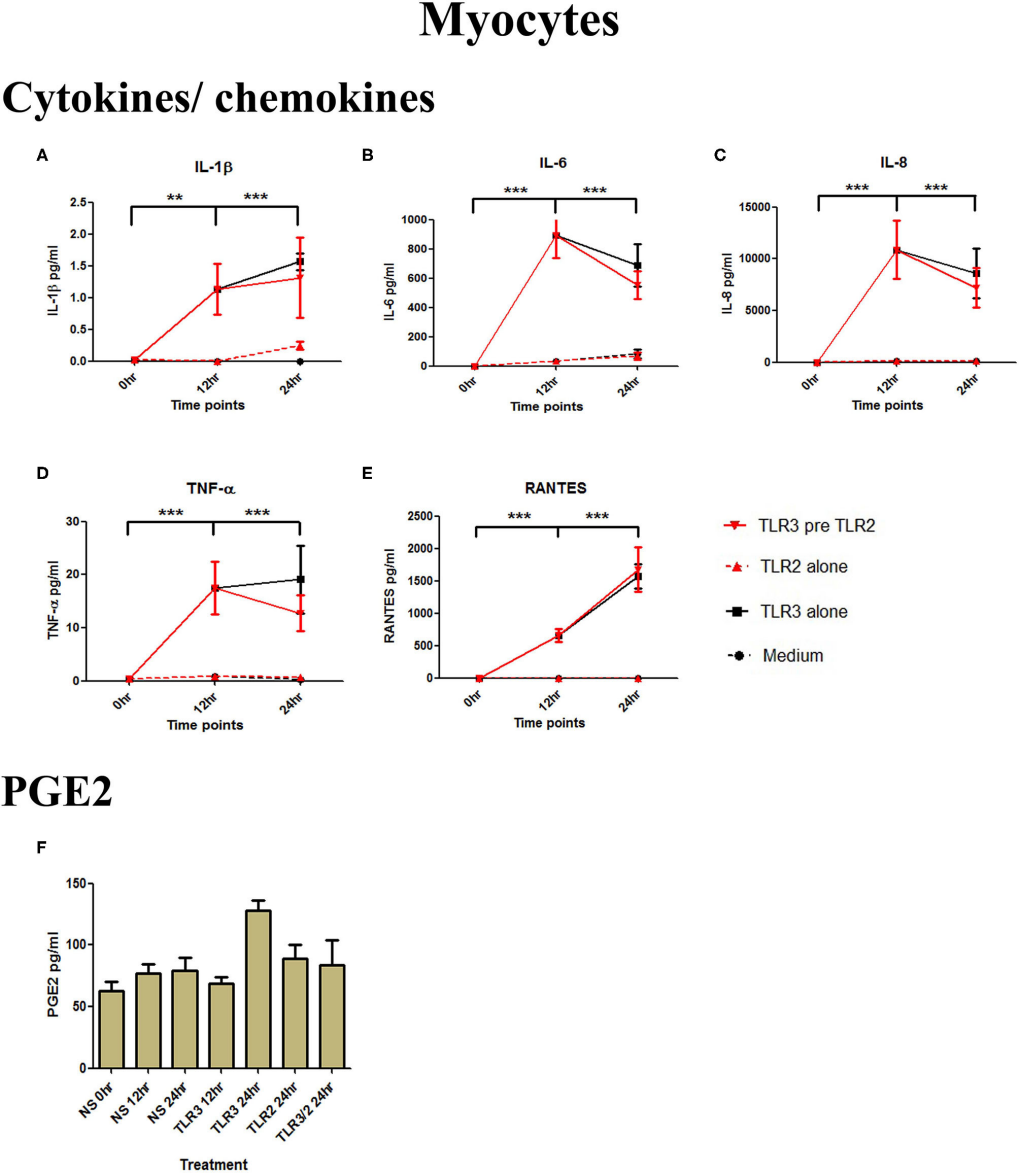
The effect of TLR3 viral priming on TLR2 agonist induced pro-inflammatory and pro-labour mediators in myocytes. Monolayer cells were primed with 25 μg/ml of poly I:C for 12 h prior to 10^5^ cells/ml of HKLM for 24 h. Supernatant was collected to quantify the pro-inflammatory and pro-labour mediators. Poly I:C stimulation alone significantly increased the production of IL-1ß, IL-6, IL-8, TNF-α, and RANTES. TLR3 priming prior to HKLM stimulation showed no augmentation in IL-1ß **(A)**, IL-6 **(B)**, IL-8 **(C)**, TNF-α **(D)**, RANTES **(E)**, and PGE2 **(F)**. For statistical analysis Two-way ANOVA with Bonferroni's multiple comparison test was used. ^*^ = effect of TLR3 compared to 0 h. ^**^ = *p* < 0.01, ^***^ = *p* < 0.001.

Although poly I:C significantly increased the production of IL-1β, IL-6, IL-8, TNF-α, and RANTES (*p* < 0.001) in myocytes, there was no increase in cells treated with 10^8^ cells/ml of HKLM alone, and no augmented production with combined TLR3/TLR2 treatment (Figures 4A–E). No increase in PGE2 occurred with either treatment combination in myocytes (Figure 4F).

#### TLR3 Priming Prior to TLR 2/6 Agonist Treatment in a Model of Ascending Infection

**Figure 5:**
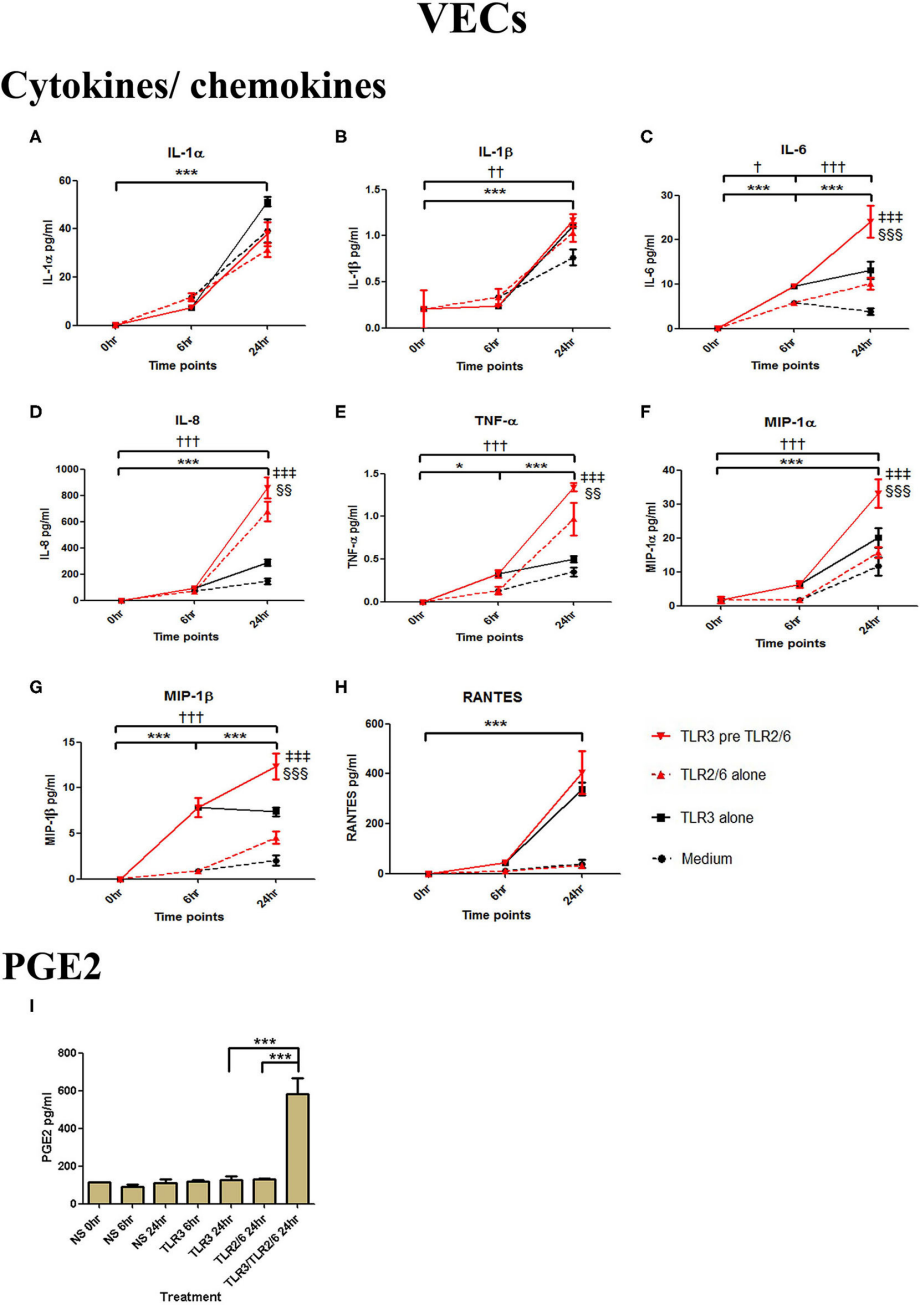
The effect of TLR3 viral priming on TLR2/6 agonist induced pro-inflammatory and pro-labour mediators in vaginal epithelial cells. Monolayer VECs were primed with 25 μg/ml of poly I:C for 6 h prior to 0.01 μg/ml of FSL-1 for 24 h and the supernatant was collected to quantify the pro-inflammatory and pro-labour mediators. Poly I:C and FSL-1 stimulation alone significantly increased the production of the cytokines and chemokines. TLR3 priming prior to FSL-1 stimulation showed no augmentation in IL-1α **(A)** and IL-1ß **(B)**. However, significant increase was observed in IL-6 **(C)**, IL-8 **(D)**, TNF-α **(E)**, MIP-1α **(F)**, and MIP-1ß **(G)**. No significant increase was observed in RANTES **(H)** chemokine. Pro-labour PGE2 **(I)** showed significant augmentation compared to TLR3 and TLR2/6 agonist stimulation alone. For statistical analysis Two-way ANOVA with Bonferroni's multiple comparison test was used. ^*^ = effect of TLR3 compared to 0 h, ^*†*^ = effect of TLR2/6 compared to 0 h, ‡ = effect of TLR3 pre TLR2/6 compared to TLR3 alone, § = effect of TLR3 pre TLR2/6 compared to TLR2/6 alone. ^*††*^ = *p* < 0.01, ^*†††*^ = *p* < 0.001, ^***^ = *p* < 0.001, §§ = *p* < 0.01, §§§ = *p* < 0.001, ‡‡‡ = *p* < 0.001.

VECs were treated with 25 μg/ml of poly I:C or vehicle for 6 h prior to treatment with 0.01 μg/ml of FSL-1 or vehicle. The TLR3 agonist poly I:C alone significantly increased the production of IL-1α, IL-1β, IL-6, IL-8, TNF-α, and the chemokines MIP-1α, MIP-1β, and RANTES (*p* < 0.001) (Figures 5A–H). FSL-1 alone also lead to a significant increase in IL-6, IL-8, TNF-α, MIP-1α, and MIP1-β. However, a further augmented response was seen in cells pre-treated with poly I:C in the production of in IL-6 (*p* < 0.001), IL-8, TNF-α (*p* < 0.01), MIP-1α, and MIP-1β (*p* < 0.001) (Figures 5C–G). Additionally, an augmented response was seen in the production of PGE2 (*p* < 0.001) (Figure 5I).

**Figure 6:**
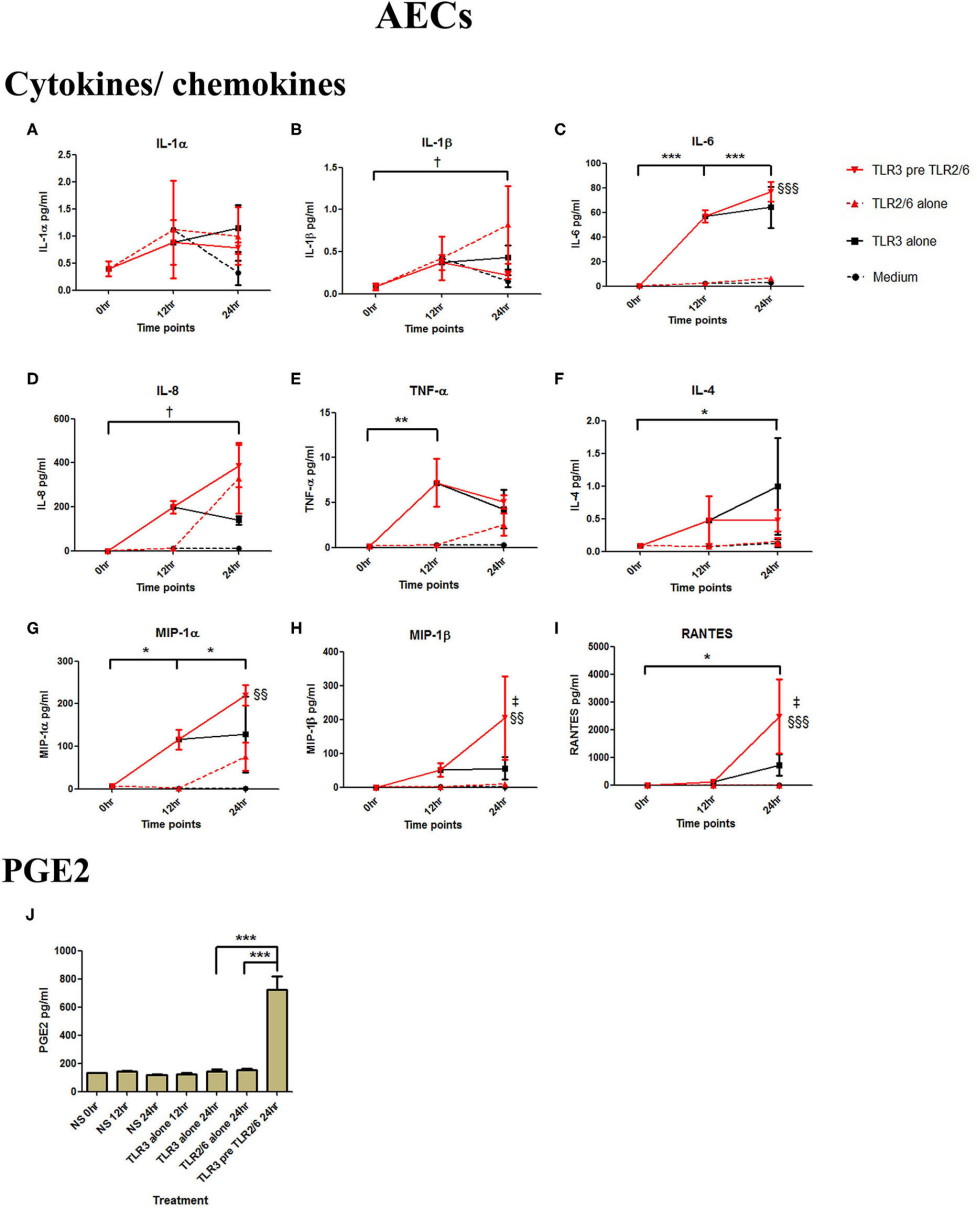
The effect of TLR3 viral priming on TLR2/6 agonist induced pro-inflammatory and pro-labour mediators in amnion epithelial cells. Monolayer cells were primed with 25 μg/ml of poly I:C for 12 h prior to 0.01 μg/ml of FSL-1 for 24 h and the supernatant was collected to quantify the pro-inflammatory and pro-labour mediators. Poly I:C and FSL-1 stimulation alone significantly increased the production of the cytokines and chemokines. TLR3 priming prior to FSL-1 stimulation showed no augmentation in IL-1α **(A)** and IL-1ß **(B)**. However, significant increase was observed in IL-6 **(C)**. No augmentation was observed with priming in IL-8 **(D)**, TNF-α **(E)** or IL-4 **(F)**. Chemokines MIP-1α **(G)**, MIP-1ß **(H)** and RANTES **(I)** showed significant augmentation with priming. Pro-labour PGE2 **(J)** also showed significant augmentation compared to TLR3 and TLR2/6 agonist stimulation alone. For statistical analysis Two-way ANOVA with Bonferroni's multiple comparison test was used. ^*^ = effect of TLR3 compared to 0 h, *^†^* = effect of TLR2/6 compared to 0 h, ‡ = effect of TLR3 pre TLR2/6 compared to TLR3 alone, § = effect of TLR3 pre TLR2/6 compared to TLR2/6 alone. ^**^ = *p* < 0.01, ^***^ = *p* < 0.001, §§ = *p* < 0.01, §§§ = *p* < 0.001.

**Figure 7:**
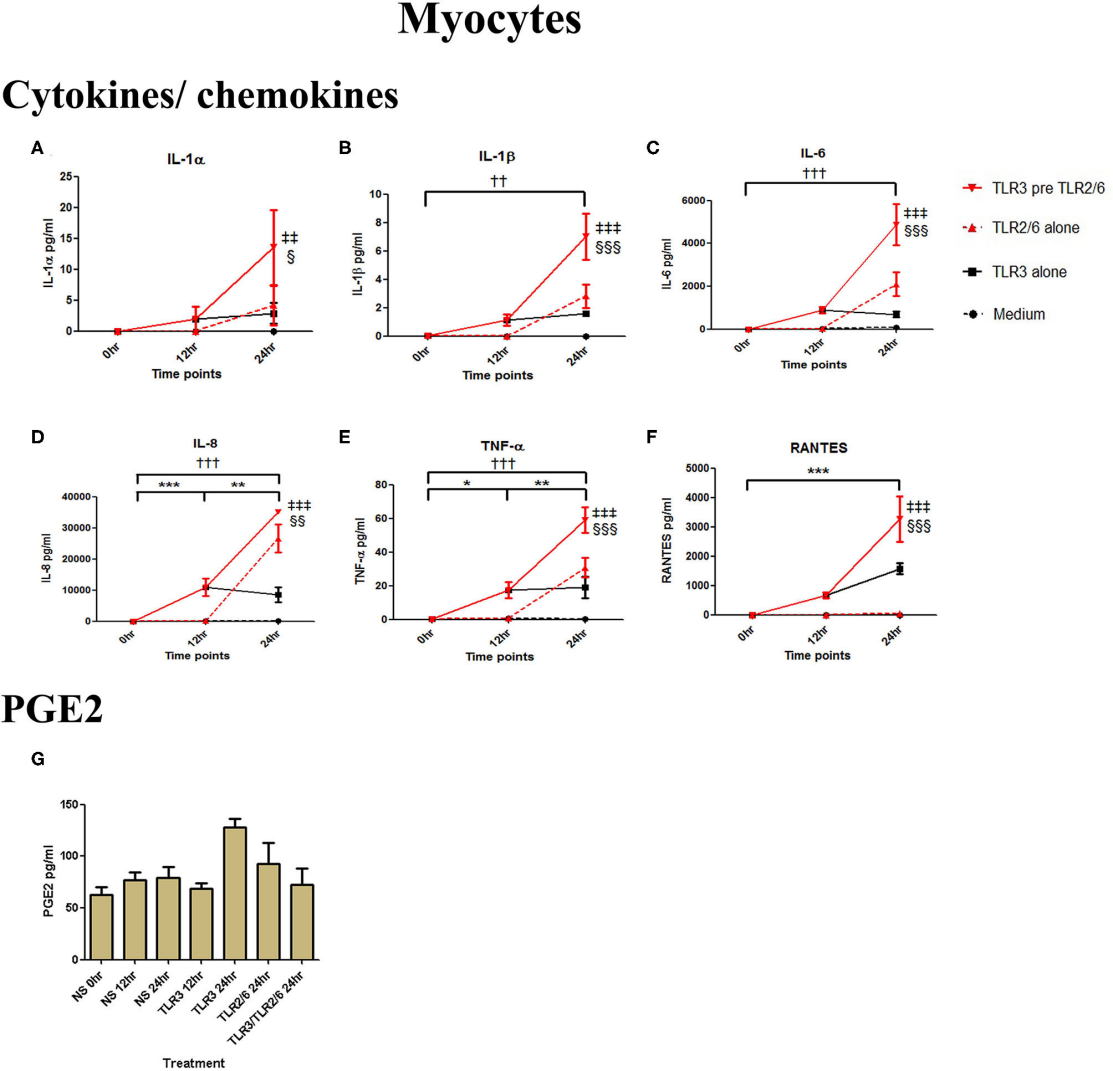
The effect of TLR3 viral priming on TLR2/6 agonist induced pro-inflammatory and pro-labour mediators in myocytes. Monolayer cells were primed with 25 μg/ml of poly I:C for 12 h prior to 0.1 μg/ml of FSL-1 for 24 h. Supernatant was collected to quantify the pro-inflammatory and pro-labour mediators. Poly I:C and FSL-1 stimulation alone significantly increased the production of the cytokines and chemokines. TLR3 priming prior to FSL-1 stimulation showed significant augmentation in IL-1α **(A)**, IL-1ß **(B)**, IL-6 **(C)**, IL-8 **(D)**, TNF-α **(E)**, and RANTES **(F)**. However, no augmentation was observed in pro-labour PGE2 **(G)**. For statistical analysis Two-way ANOVA with Bonferroni's multiple comparison test was used. ^*^ = effect of TLR3 compared to 0 h, ^*†*^ = effect of TLR2/6 compared to 0 h, ‡ = effect of TLR3 pre TLR2/6 compared to TLR3 alone, § = effect of TLR3 pre TLR2/6 compared to TLR2/6 alone. ^*††*^ = *p* < 0.01, ^*†††*^ = *p* < 0.001, ^**^ = *p* < 0.01, ^***^ = *p* < 0.001, §§ = *p* < 0.01, §§§ = *p* < 0.001, ‡‡ = *p* < 0.01, ‡‡‡ = *p* < 0.001.

Poly I:C (25 μg/ml, 12 h) treatment increased the production of IL-6 (*p* < 0.001), TNF-α (*p* < 0.01), MIP-1α, and RANTES (*p* < 0.01), and IL-4 (*p* < 0.05) in AECs, and FSL-1 (0.01 μg/ml) treatment alone also increased concentrations of IL-1β and IL-8 (*p* < 0.05) (Figure 6). However, where cells were primed with poly I:C prior to FSL-1, there was an increase in the concentrations of IL-6 (*p* < 0.001), MIP-1α, and MIP-1β (*p* < 0.01) and RANTES (*p* < 0.001). A synergistic increase in of PGE2 (*p* < 0.001), with TLR3/TLR2/6 treatment was also seen in AECs (Figure 6J). Similarly, in myocytes, a synergistic increase in IL-1α (*p* < 0.05), IL-1β, IL-6 (*p* < 0.001), IL-8 (*p* < 0.01), TNF-α, RANTES (*p* < 0.001) was seen with poly I:C priming prior to treatment with FSL-1 (0.1 μg/ml) (Figures 7A–G).

### TLR3-Induced Activation of NF-κB, AP-1, and IRF in PBMCs and Placenta Explants

**Figure 8:**
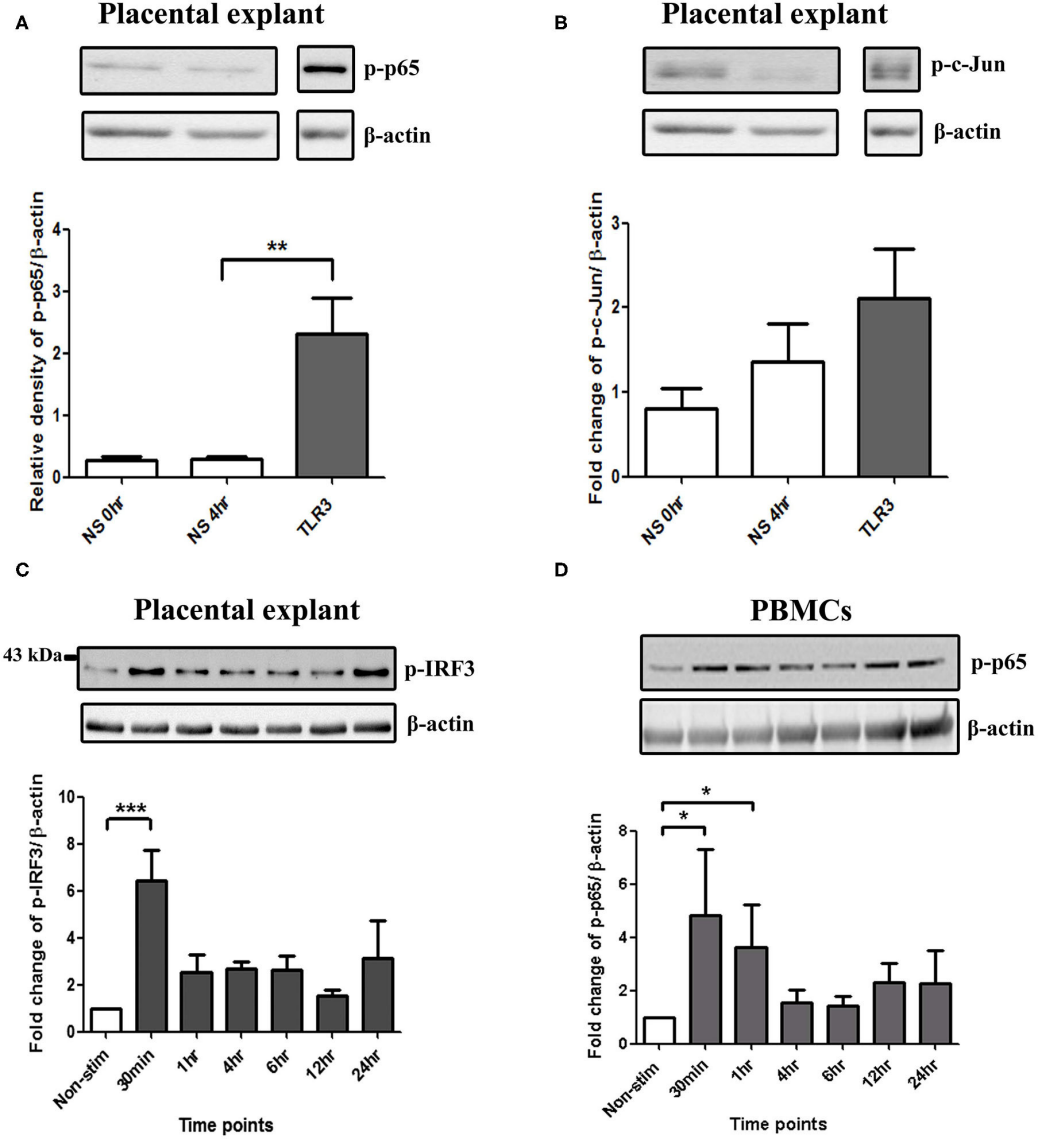
Activation of NF-κB in PBMCs, and NF-κB, AP-1, and IRF in placental explants. An increase in p-p65 was seen after 4 h of treatment with 25 μg/ml poly I:C in placental explants (*p* < 0.01) **(A)** but no significant increase was observed in p-c-Jun **(B)**. Early activation of IRF3 at 30 min (*p* < 0.001) was seen in placental explants **(C)**. PBMCs were treated with 5 μg/ml of Poly I:C for 30 min, 1 h, 4 h, 6 h, 12 h, and 24 h. A significant increase in phospho-p-65 was seen by 30 min (*p* < 0.05) **(D)**. For statistical analysis, One-way ANOVA was used for normally distributed data and Kruskal-Wallis was used for data not normally distributed with Dunnett's multiple comparison test *n* = 4. ^*^ = *p* < 0.05, ^**^ = *p* < 0.01, ^***^ = *p* < 0.001.

In placental explants, Poly I:C led to a significant increase in p-p65 at 4 h and p-IRF3 at 30 min (*p* < 0.01 and *p* < 0.001), with no significant increase in p-c-Jun observed (Figures 8A–C, Supplementary Figure 3). in PBMCs, A significant increase in p-p65 was seen with poly I:C incubation at 30 min and 1 h (*p* < 0.05) (Figure 8D). However, p-c-Jun or p-IRF3 levels were too low for detection in PBMCs.

### A Model of Haematogenous Spread of Infection: Placental Explants Show an Enhanced Anti-inflammatory Response With TLR3 Agonist Viral Priming Prior to TLR2 and TLR2/6 Agonist Treatment

To mimic the effect of haematogenous infection, PBMCs and placental explants were incubated with the same TLR agonists used in the model of ascending infection above. HKLM led to an increase in NF-κB activation in PBMCs and p-c-Jun in placental explants, however no significant increase was seen with FSL-1 treatment (Supplementary Figures 1, 3).

**Figure 9:**
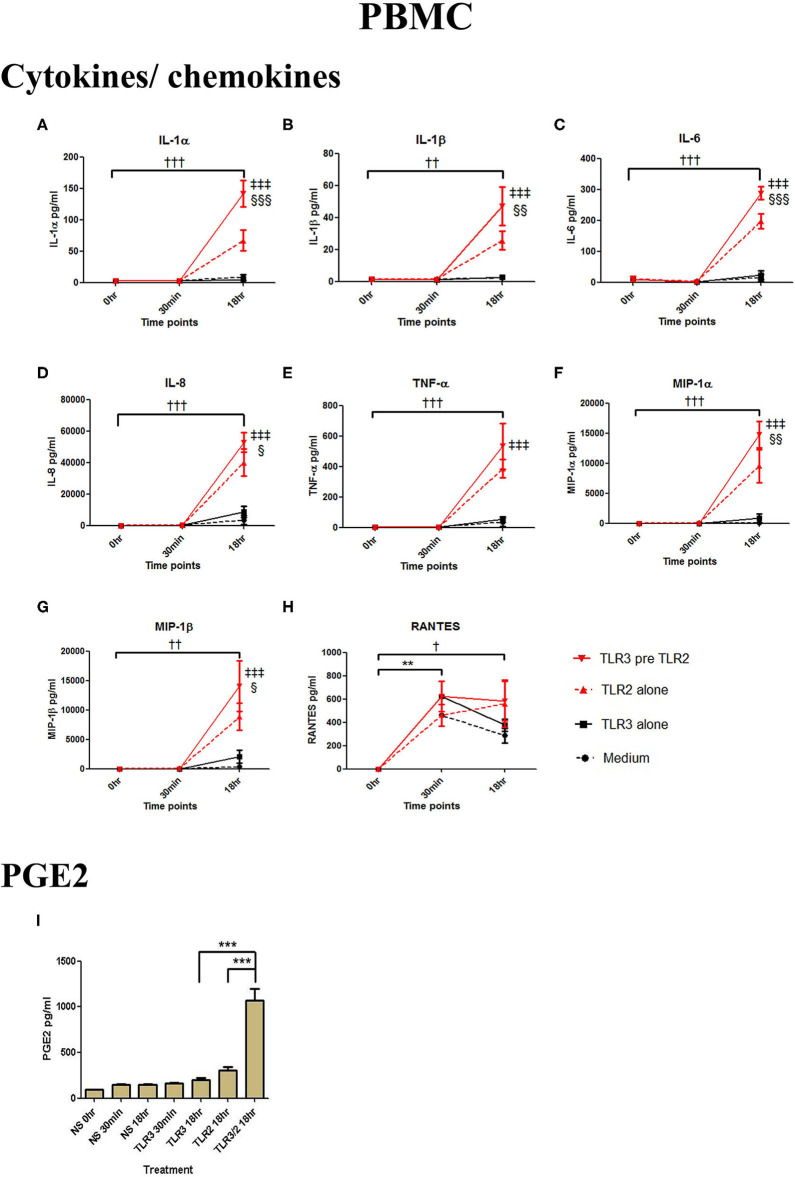
The effect of TLR3 viral priming on TLR2 agonist induced pro-inflammatory and pro-labour mediators in PBMCs. PBMCs were primed with 5 μg/ml of poly I:C for 30 min prior to 10^8^ cell/ml of HKLM for 18 h. Supernatant was collected to quantify the pro-inflammatory and pro-labour mediators. Poly I:C and HKLM stimulation alone significantly increased the production of the RANTES chemokines. TLR3 priming prior to HKLM stimulation showed a significant augmentation in IL-1α **(A)**, IL-1ß **(B)**, IL-6 **(C)**, and IL-8 **(D)**. No significant augmentation was observed in TNF-α. **(E)** Chemokines MIP-1α **(F)** and MIP-1ß **(G)** showed significant augmentation but not RANTES **(H)**. Significant augmentation was also observed in pro-labour PGE2 **(I)**. For statistical analysis Two-way ANOVA with Bonferroni's multiple comparison test was used. ^*^ = effect of TLR3 compared to 0 h, ^*†*^ = effect of TLR2 compared to 0 h, ‡ = effect of TLR3 pre TLR2 compared to TLR3 alone, § = effect of TLR3 pre TLR2 compared to TLR2 alone. ^*††*^ = *p* < 0.01, ^*†††*^ = *p* < 0.001, ^**^ = *p* < 0.01, ^***^ = *p* < 0.001, §§ = *p* < 0.01, §§§ = *p* < 0.001, ‡‡ = *p* < 0.01, ‡‡‡ = *p* < 0.001.

**Figure 10:**
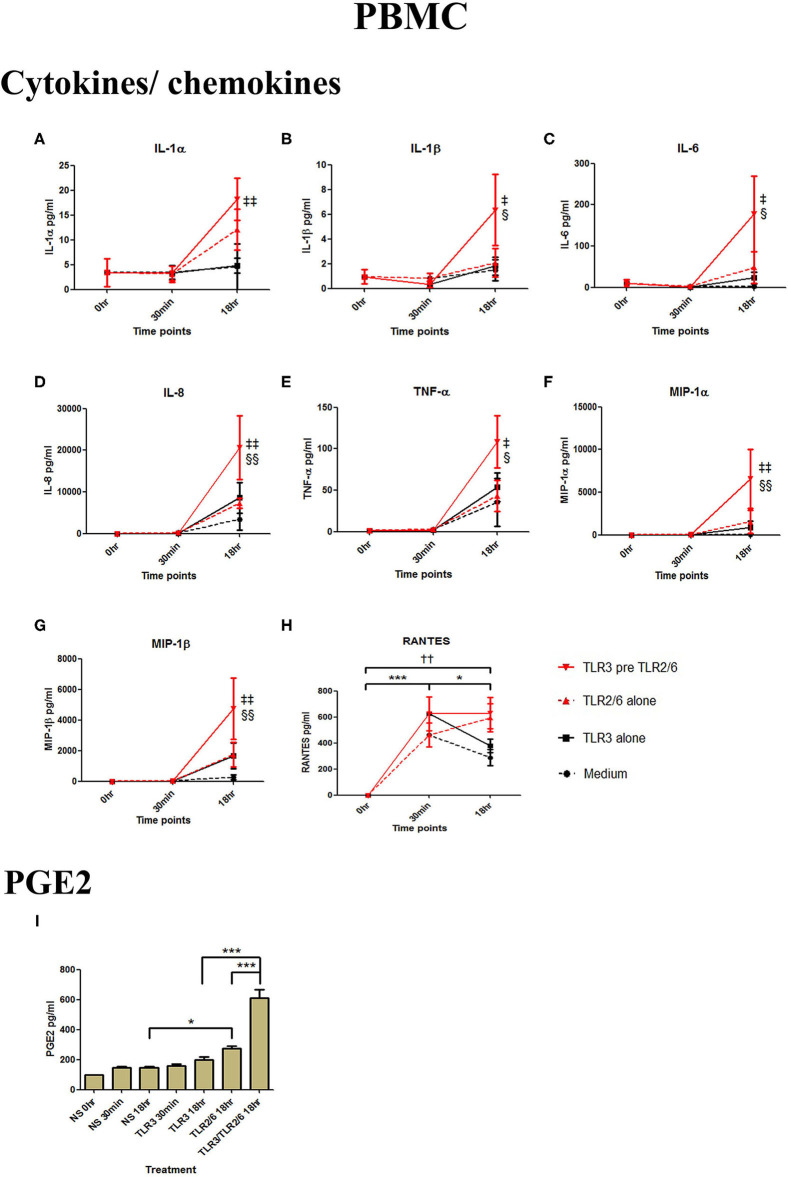
The effect of TLR3 viral priming on TLR2/6 agonist induced pro-inflammatory and pro-labour mediators in PBMCs. PBMCs were primed with 5 μg/ml of poly I:C for 30 min prior to 0.1 μg/ml of FSL-1 for 18 h. Supernatant was collected to quantify the pro-inflammatory and pro-labour mediators. TLR3 and FSL-1 stimulation alone only significantly increased the production of RANTES. TLR3 priming prior to FSL-1 stimulation showed no augmentation on IL-1α but a significant augmentation in IL-1ß **(B)**, IL-6 **(C)** and IL-8 **(D)**, TNF-α **(E)**, MIP-1α **(F)**, and MIP-1ß **(G)**. However, no significant augmentation was observed in RANTES **(H)**. Significant augmentation was also observed in pro-labour PGE2 **(I)**. For statistical analysis Two-way ANOVA with Bonferroni's multiple comparison test was used. ^*^ = effect of TLR3 compared to 0 h, ^*†*^ = effect of TLR2/6 compared to 0 h, ‡ = effect of TLR3 pre TLR2/6 compared to TLR3 alone, § = effect of TLR3 pre TLR2/6 compared to TLR2/6 alone. ^*††*^ = *p* < 0.01, ^*^ = *p* < 0.05, ^***^ = *p* < 0.001, § = *p* < 0.05, §§ = *p* < 0.01, ‡ = *p* < 0.05, ‡‡ = *p* < 0.01.

In PBMCs, HKLM significantly increased the production of IL-1α, IL-1β, IL-6, IL-8, TNF-α, and the chemokines MIP-1α, MIP-1β and RANTES, but poly I:C only increased the production of RANTES (Figures 7A–H). However, priming with poly I:C prior to HKLM treatment led to a synergistic increase in IL-1α (*p* < 0.001), IL-1β (*p* < 0.01), IL-6 (*p* < 0.001), IL-8 (*p* < 0.05), MIP-1α (*p* < 0.01), and MIP-1β (*p* < 0.05) and PGE2 (*p* < 0.001) (Figures 9A–I). FSL-1 treatment alone did not change cytokine concentrations in PBMCs apart from RANTES, which increased in response to both FSL-1 and poly I:C treatment alone (*p* < 0.05 and *p* < 0.01, respectively; Figure 10). However, a synergistic effect was seen on pro-inflammatory and pro-labour mediator production with poly I:C priming prior FSL-1 treatment in PBMCs including IL-1β, IL-6 (*p* < 0.05), IL-8, MIP-1α, and MIP-1β and PGE2, but not IL-1α (Figures 10A–I).

**Figure 11:**
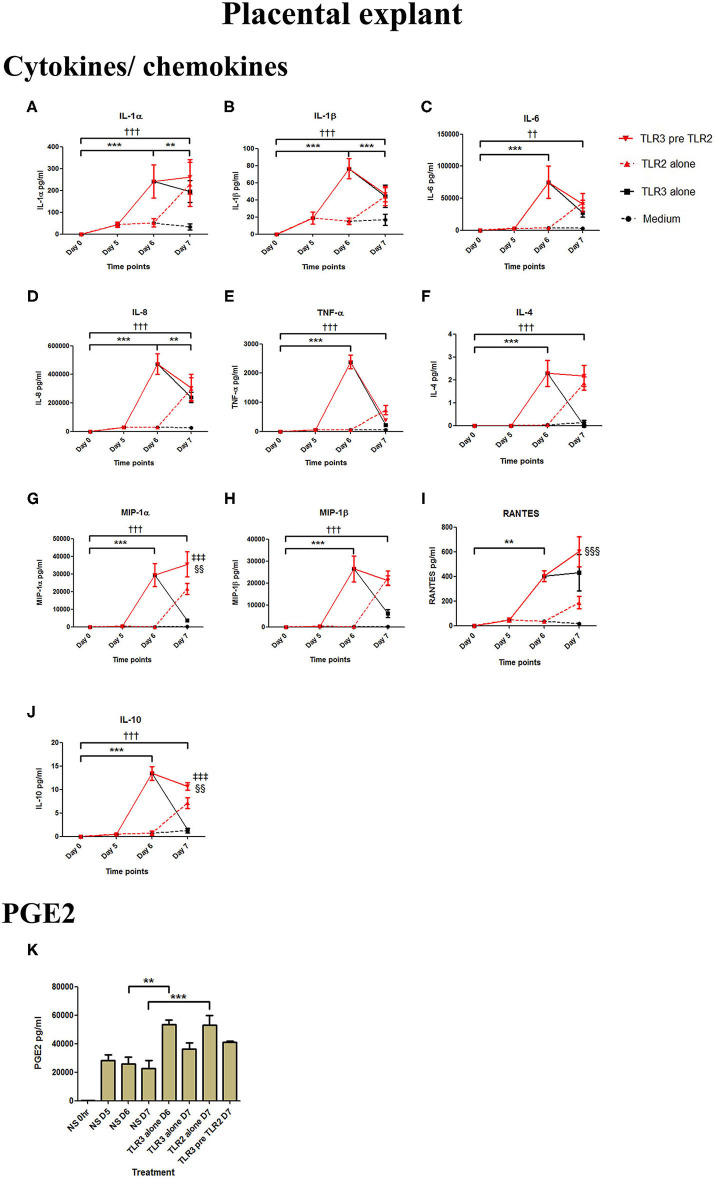
The effect of TLR3 viral priming on TLR2 agonist induced pro-inflammatory and pro-labour mediators in placental explants. Explants were primed with 25 μg/ml of poly I:C for 24 h prior to 10^8^ cells/ml of HKLM for 24 h. Supernatant was collected to quantify the pro-inflammatory and pro-labour mediators. Poly I:C and HKLM stimulation alone significantly increased the production of the cytokines, chemokines and PGE2. TLR3 priming prior to HKLM stimulation showed no augmentation in IL-1α **(A)**, IL-1ß **(B)**, IL-6 **(C)** IL-8 **(D)** TNF-α **(E)**, IL-4 **(F)** or MIP-1ß **(H)**. A significant augmentation was only observed in MIP-1α **(G)** RANTES **(I)** and the anti-inflammatory cytokine IL-10 **(J)**. No augmentation was observed in pro-labour PGE2 **(K)**. For statistical analysis Two-way ANOVA with Bonferroni's multiple comparison test was used. ^*^ = effect of TLR3 compared to 0 h, ^*†*^ = effect of TLR2 compared to 0 h, ‡ = effect of TLR3 pre TLR2 compared to TLR3 alone, § = effect of TLR3 pre TLR2 compared to TLR2 alone. ^**^ = *p* < 0.01, ^***^ = *p* < 0.001, ^*††*^ = *p* < 0.01, ^*†††*^ = *p* < 0.001, §§ = *p* < 0.01, §§§ = *p* < 0.001.

**Figure 12:**
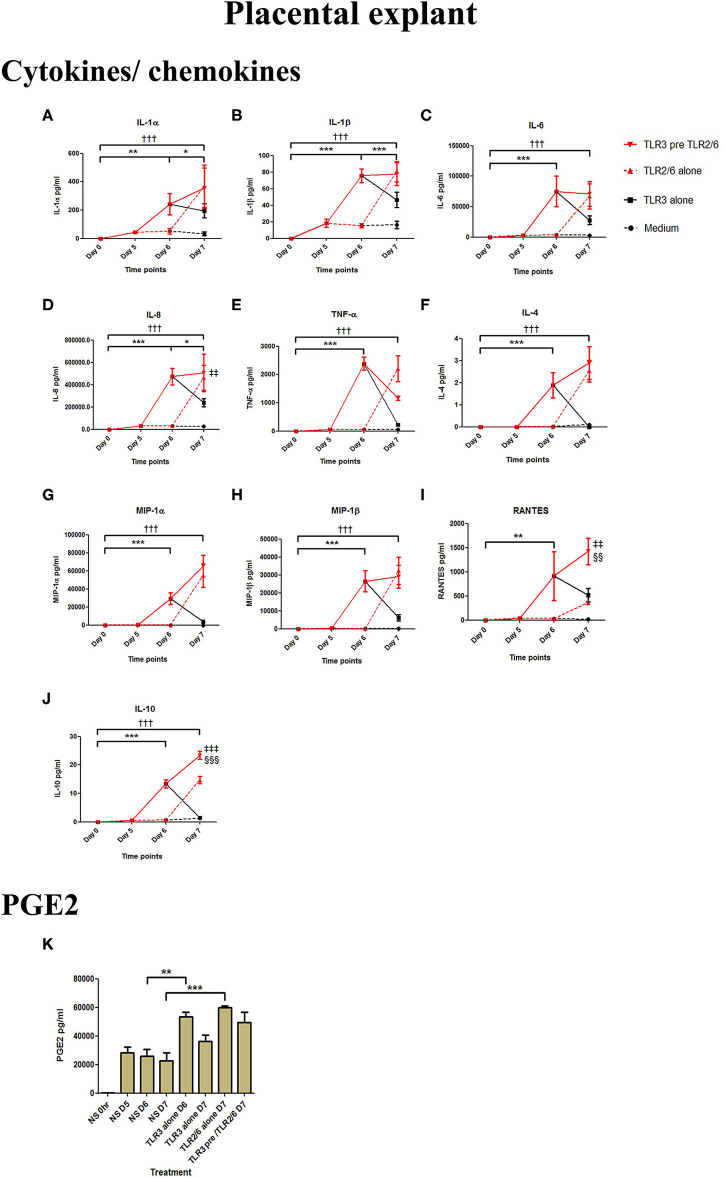
The effect of TLR3 viral priming on TLR2/6 agonist induced pro-inflammatory and pro-labour mediators in placental explants. Explants were primed with 25 μg/ml of poly I:C for 24 h prior to 0.1 μg/ml of FSL-1 for 24 h. Supernatant was collected to quantify the pro-inflammatory and pro-labour mediators. Poly I:C and FSL-1 stimulation alone significantly increased the production of the cytokines, chemokines and PGE2. TLR3 priming prior to FSL-1 stimulation showed no augmentation in IL-1α **(A)**, IL-1ß **(B)**, IL-6 **(C)**, IL-8 **(D)**, TNF-α **(E)**, IL-4 **(F)**, MIP-1α **(G)**, MIP-1ß **(H)**. However, significant augmentation was observed in RANTES **(I)** and anti-inflammatory IL-10 **(J)**. No augmentation was observed in pro-labour PGE2 **(K)**. For statistical analysis Two-way ANOVA with Bonferroni's multiple comparison test was used. ^*^ = effect of TLR3 compared to 0 h, ^*†*^ = effect of TLR2/6 compared to 0 h, ‡ = effect of TLR3 pre TLR2/6 compared to TLR3 alone, § = effect of TLR3 pre TLR2/6 compared to TLR2/6 alone. ^**^ = *p* < 0.01, ^***^ = *p* < 0.001, ^*†††*^ = *p* < 0.001, §§ = *p* < 0.01, §§§ = *p* < 0.001, ‡‡ = *p* < 0.01, ‡‡‡ = *p* < 0.001.

Poly I:C treatment of placental explants led to a significant increase in the production of IL-1α, IL-1β, IL-6, IL-8, TNF-α, IL-4, and the chemokines MIP-1α, MIP-1β (*p* < 0.001), and RANTES, and PGE2 (*p* < 0.01) (Figures 11A–K, 12A–K). HKLM incubation alone led to the significant increase of all inflammatory mediators except for RANTES (Figure 11I). However, with priming, an augmented response was only seen in the production of IL-10 (*p* < 0.01), MIP-1α (*p* < 0.01), and RANTES (*p* < 0.001). Similarly, although FSL-1 incubation alone increased the production of IL-1α, IL-1β, IL-6, IL-8, TNF-α, IL-4, and the chemokines MIP-1α, MIP-1β, and PGE2, priming did not lead to a further augmentation in pro-inflammatory/pro-labour mediators, but instead led to a significant increase in IL-10 (*p* < 0.001) and RANTES (*p* < 0.01) (Figure 12).

### PBMC-Derived Inflammatory Mediators From TLR3 Primed and TLR2/6 Treated Cells Induce an Augmented Pro-inflammatory Response in Placental Explants

**Figure 13:**
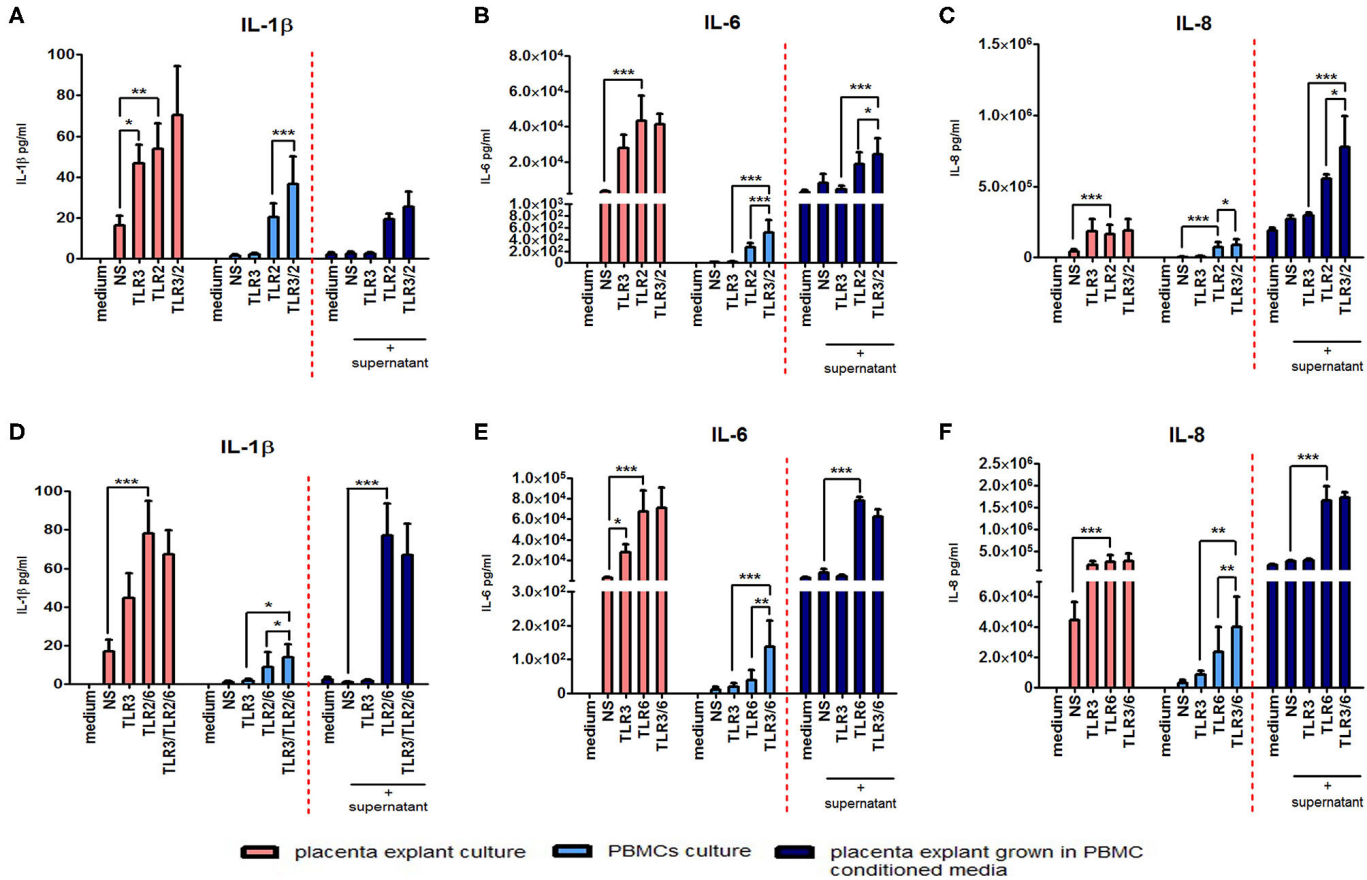
The effect of co-culturing placental explants with PBMC conditioned media on IL-1ß, IL-6 and IL-8. Placental explants were cultured and treated with media derived from TLR3-primed or TLR2 or TLR2/6 or TLR3 agonist treated PBMCs for 24 h. Supernatant was collected and the level of pro-inflammatory cytokines IL-1ß, IL-6, and IL-8 production was determined using ELISA. Pink-coloured histograms demonstrate placental explant priming experiment results, showing no augmentation of the cytokine production with TLR3/TLR2 treatment **(A–C)** or TLR3/ and TLR2/6 treatment **(D–F)**. Light blue-coloured histograms IL concentrations from PBMCs following TLR3/TLR2 treatment **(A–C)** or TLR3/ and TLR2/6 treatment **(D–F)**. Cytokine concentrations from culturing placental explants in PBMC conditioned media is represented in the dark blue-coloured histograms for TLR3/TLR2 treatment **(A–C)** or TLR3/ and TLR2/6 treatment **(D–F)**. No augmentation was seen with IL-1ß **(A)** but a significant augmented production of IL-6 **(B)** and IL-8 **(C)** was observed compared to placental explants cultured in conditioned media from PBMCs-treated with TLR3 and TLR2 agonists alone. Treatment of placental explants with media derived from PBMCs treated with TLR3 and TLR2/6 agonist media showed no augmentation of IL-1ß **(D)**, IL-6 **(E)**, or IL-8 **(F)**. For statistical analysis, Two-way ANOVA with Bonferroni's multiple comparison test was used *N* = 4. ^*^ = *p* < 0.05, ^**^ = *p* < 0.01, ^***^ = *p* < 0.001.

The anti-inflammatory response of placental explants to TLR2/6 with TLR3 priming led us to test the immunomodulatory effect of PBMC conditioned media on the placental explants as a model systemic inflammation. Cytokine concentrations were measured from explant cultures treated with the TLR agonists (pink histograms) or PBMC derived supernatant (light blue histograms), or from placental explants cultured in PBMCs conditioned media (dark blue histograms) (Figure 13). Incubation of placental explants with conditioned medium from PBMCs treated with poly I:C and HKLM led to a synergistic increase in IL-6 (*p* < 0.05) and IL-8 (*p* < 0.05), but not IL-1β (Figures 13A–C). However, no synergistic increase was seen in cytokine production from placental explants grown in conditioned medium from PBMCs treated with poly I:C and FSL-1 (Figures 13D–F).

### The TLR3 Agonist Poly I:C Increases the Expression of Both TLR3 and TLR2 Receptors, but Not of the TLR6 Receptor

**Figure 14:**
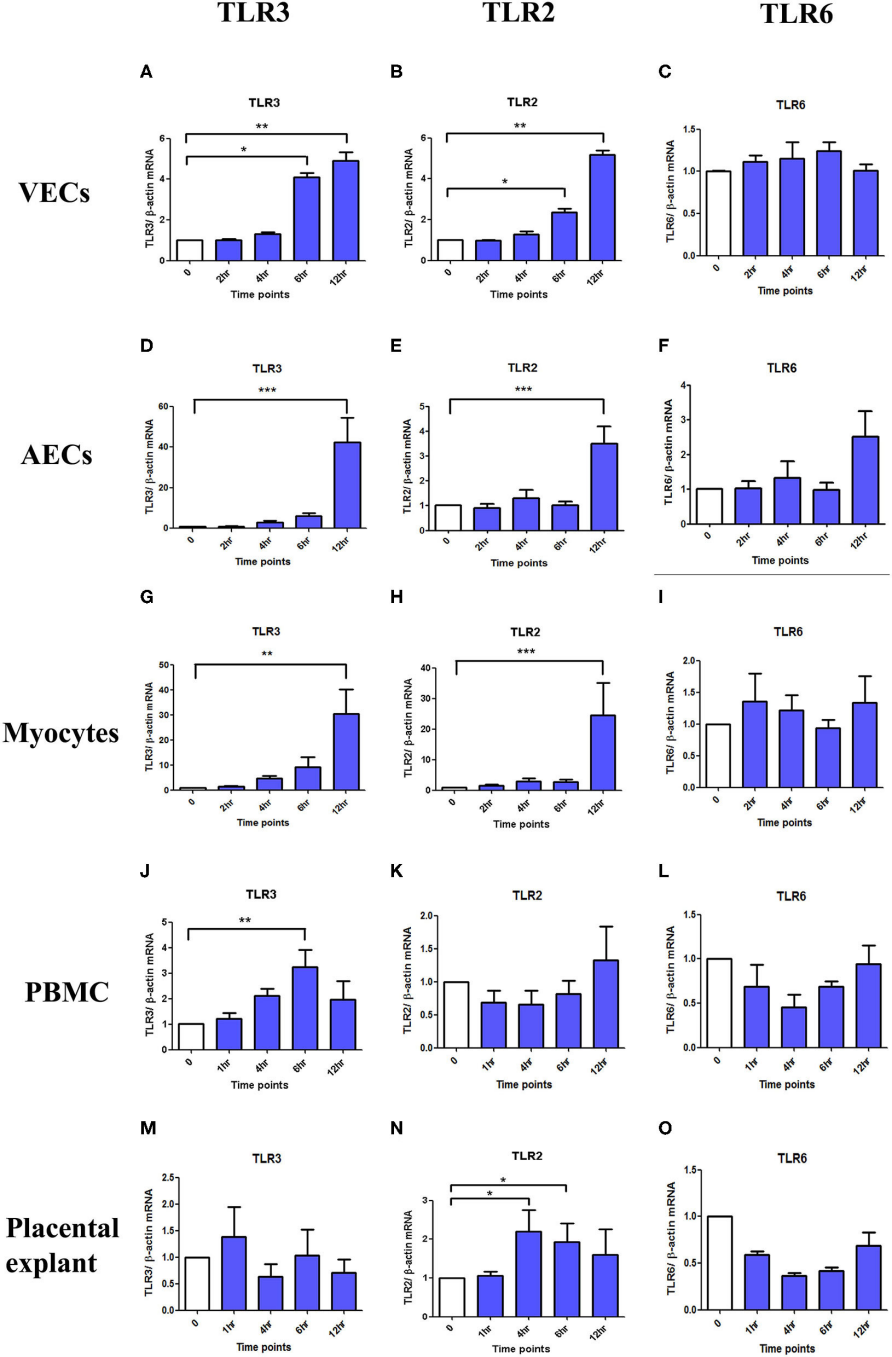
The effect of TLR3, 2 and 6 mRNA expression with poly I:C stimulation in VECs, AECs, myocytes, PBMC and placental explants. VECs, AECs, myocytes, and placental explant were treated with 25 μg/ml while PBMCs were treated with 5 μg/ml of poly I:C. The level of the mRNA expression was determined after 2, 4, 6, and 12 h in VEC, amniocytes and myocytes while at 1, 4, 6, and 12 h in PBMC and placental explant using ß-actin as the loading control. Total RNA was extracted and RT-QPCR was performed to quantify TLR3, 2, and 6 mRNA level. In VEC, amniocytes and myocytes, TLR3 **(A,D,G)** and TLR2 **(B,E,H)** are significantly increased after poly I:C stimulation, however, no increased was observed in TLR6 **(C,F,I)**. In PBMC, only TLR3 is significantly increased **(J)** but a non- significant increase was observed with TLR2 **(K)** and TLR6 **(L)**. In contrast to PBMC, in placental explant, no significant increase was observed in TLR3 **(M)**, but a significant increase was observed in TLR2 **(N)**. No change was observed in TLR6 mRNA expression **(O)**. For statistical analysis Kruskal-Wallis with Dunnett's multiple comparison test was used. A minimum of *n* = 3 biological replicates were used. ^*^ = *p* < 0.05, ^**^ = *p* < 0.01, ^***^ = *p* < 0.001.

TLR3 and TLR2 mRNA expression were significantly increased after poly I:C stimulation in VECs (*p* < 0.05 at 6 h, *p* < 0.01 at 12 h), AECs (*p* < 0.001 at 12 h), and myocytes (TLR3; *p* < 0.01 at 12 h and TLR2; *p* < 0.001 at 12 h) (Figures 14A–I). In PBMC, only TLR3 mRNA (*p* < 0.01 at 6 h) was significantly increased, although a non-significant increase was seen in TLR2 expression at 12 h (Figures 14J,K). No differences were seen in TLR6 expression in any of the cell types (Figures 14C,F,I,L). No changes in TLR3 or 6 expression were seen in placental explants, but there was a transient increase in TLR2 expression at 4 and 6 h (*p* < 0.05), which was lost by 12 h (Figures 14M–O).

## Discussion

There is an established causal link between infection/inflammation and PTB (7) and growing evidence for a role for the vaginal microbiota in shaping PTB risk (20–23). Although certain bacterial species are associated with PPROM and sPTL, clinical use of antibiotics does not mitigate risk, and not all women with an adverse vaginal microbial composition deliver preterm. Other factors must therefore be involved, and one plausible contributor is clinical or subclinical viral infection. Animal studies have shown that viral infection modulates host immune response, increasing susceptibility to bacterial induced sPTL. However, there are limited studies in the human examining this double hit effect. To address this, we considered two possible routes by which viral and bacterial stimuli might impact the maternal-fetal interface and myometrium. In this study, VECs, AECs and myocytes were used to represent the target cells in an *in vitro* model of ascending infection, and PBMCs and placental explants to represent the target cells in a model of systemic inflammation and haematogenous infection. We demonstrate that viral priming prior to incubation with bacterial products leads to a synergistic increase in pro-inflammatory and pro-labour mediators which is more pronounced in our model of ascending infection.

Systemic viral infections such as human immunodeficiency virus (HIV) (55), Hepatitis B (56) and influenza have all been linked to higher rates of sPTL. Insight from epidemiological studies following the H1N1 pandemic also reported on higher rates of sPTL in women with underlying health conditions (57), and lower rates of preterm delivery in women who had been vaccinated (58). In support of the role of haematogenous spread of viral infection in preterm birth, a study of 71 preterm and 122 full term placentas demonstrated a greater proportion of adenovirus positive placentas in preterm deliveries and in cases with histological evidence of chorioamnionitis (46). Although a significant number of viral taxa have been detected in amniotic fluid, only a few such as herpes simplex virus, adenovirus, enterovirus and cytomegalovirus have been linked to PTB (59). Viral infections of the lower reproductive tract can also predispose to PTB, with several studies implicating cervical human papillomavirus (HPV) (60, 61) and herpes simplex virus (HSV) (62, 63) in the aetiology of PTB.

Many animal studies have also provided evidence for a potential role for viruses in sPTB. Animal models investigating the role of single viral pathogens and PTB commonly use the TLR3 agonist, poly I:C. Koga et al. demonstrated a 100% sPTB rate in mice treated with intraperitoneal poly I:C and a 0% rate in TLR3 knockout mice (47). However, TLR9 activation from intraperitoneal injection of CpG oligodeoxynucleotide into IL-10 deficient mice has also been reported to cause a 100% sPTB rate (64). There is also evidence that viral infection may mediate local inflammation and pro-labour changes. HSV-2 causes histological evidence of collagen remodelling and increased hyaluronic acid synthesis leading to cervical ripening, and aberrant expression of oestrogen and progesterone receptors in the cervical epithelium (65).

*In vitro* studies also support a role for viruses in immune modulation in human gestational tissues. Murine herpes virus-68 (MHV-68) stimulation of human fetal membranes causes increased production of IL-1β, IL-6, IL-8, and IFN-γ (66). Amnion, choriodecidua and placental explants are highly responsive to both the TLR3 agonist poly I:C and the TLR agonist ssRNA40, each leading to increased release of IL-6 and IL-8 (67). Several studies have also demonstrated pro-inflammatory cytokine release by cultured myocytes, vaginal and cervical epithelial cells upon incubation with poly I:C (68–72). In our study, we confirmed that the TLR3 agonist poly I:C induces inflammation in VECs, myocytes, and placental explants, and demonstrate that the effect reported in fetal membranes is reproduced at the cellular level in AECs. TLR3 recognises both virus–derived double-stranded RNA (dsRNA) (73) and its synthetic analogue poly I:C. While TLR3 activation leads to a pro-inflammatory response, it also is capable of activating an anti-viral response through the production of the Type I, II, and III interferons (74–76) as observed in cases of hepatitis B and C viruses, herpesvirus, and rotavirus (77).

Unlike other TLR family members, TLR3 is not dependant on myeloid differentiation factor 8 (MyD88) as the signalling adaptor protein (78). TLR3 mediates transduction via the adaptor protein TICAM-1/TRIF (79, 80) activating transcription factors NF-κB, AP-1, and IRF3, leading to the induction of cytokine and chemokine production (73). As anticipated, we saw a cell type dependent increase in all three transcription factors when cells were treated with poly I:C and a significant increase in phospho-IRF3 was seen in all cell types of the model of ascending infection. Activation of NF-κB in the fundal myometrium (81) and cervix (82) is strongly associated with labour onset and NF-κB activation is observed in fetal membranes prior to labour (83). The promoter region of pro-inflammatory cytokines TNF-α, IL-8, IL-6, IL-1β, OTR (28), and those of COX-2, MMP-1, and MMP-9 genes all contain binding sites for NF-κB (84) lending support to its role in uterine contractility, membrane rupture and cervical modelling. Similarly, activation of AP-1 upregulates an array of pro-inflammatory and pro-labour genes as the promoter regions for IL-8 (31), COX-2 (29, 85), oxytocin receptor (OTR) (86, 87), Cx43 and MMP-9 all contain AP-1 binding sites (86, 88). We have previously shown that AP-1 activation is a key terminal mediator of inflammation induced PTL in the mouse (29). More recently, the transcription factor IRF3 has also been highlighted as potentially playing a role in PTB. A review of studies linking genetic polymorphisms with the risk of sPTL identified IRF-3 as a strong candidate transcription factor in the aetiology of sPTB (89). Taken together, our data demonstrate the capacity for signalling via TLR3 to activate the key transcription factors known to regulate pro-labour and pro-inflammatory mediators in a situation of ascending infection, systemic inflammation and haematogenous spread of infection induced PTL.

We next explored whether activation of TLR3 leads to augmentation of the bacterial product induced inflammation in VECs, AECs and myocytes in a model of ascending infection/inflammation. We found significant synergy between TLR3 and TLR2/6 agonists in inducing cytokine, chemokine and PGE2 production (Figures 5–7, 10). Murine studies clearly demonstrate increased rates of preterm labour in response to exposure to both viral and bacterial stimuli (47, 48, 50), however most models focus on the effect of systemic infection, with few models using vaginal or intrauterine routes for administration. Racicot et al. tested the effect of systemic viral infection on local vaginal infection and demonstrated that systemic administration of MHV-68 was required to facilitate ascending infection of *U. urealyticum* from the vagina to decidua in mice (90). However, in this study, MHV-68 led to a reduction in *U. urealyticum* induced cytokine and chemokine production and in TLR 2,3 and 4 mRNA expression. Racicot and colleagues later tested the effect of vaginal HSV-2 infection on vaginally administered *E. coli* induced PTB, and showed histological evidence of cervical remodelling and a significant increase in sPTB rates with dual treatment compared to single treatment alone (65).

As has previously been reported in a study using a 3D culture model of vaginal epithelial cells (91), we found that VECs produced high concentrations of pro-inflammatory cytokines including IL6 and IL-8 in response to poly I:C (TLR3 agonist) or FSL-1 (TLR2/6 agonist) alone (Figure 5). We also demonstrated a synergistic increase when cells were primed with poly I:C. Similarly, a synergistic increase in concentrations of the chemokines MIP-1α and MIP-β, and PGE2 was seen. However, neither HKLM (TLR2 agonist) alone or in combination with poly I:C led to increases in any of the mediators (Figure 2). TLR2 forms heterodimers with TLR1 and TLR6; TLR2/1 recognises lipopeptides from Gram-negative bacteria, whereas the TLR2/6 heterodimers recognise lipopeptides from Gram-positive bacteria. FSL-1, used in our study, elicits its response via the TLR2/TLR6 heterodimer. There is an association between Gram-positive bacteria colonisation, particularly Group B *Streptococcus*, and preterm birth (92), which may be explained exclusively through promotion of increases in pro-inflammatory cytokines, as demonstrated by *in vitro* studies of cultured vaginal and cervical epithelial cells (93). However, not all women with Group B *Streptococcus* deliver preterm, therefore other factors must contribute to the pathophysiology. High diversity and instability of the bacterial vaginal communities are associated with sPTB (94, 95). Consistent with our findings, recent data suggests having both high bacterial diversity and a high viral diversity in early pregnancy is associated with an even higher risk (96), supporting a mechanism for multi-pathogen induced preterm labour in some women.

As with VECs, we also saw an increase in cytokine, chemokine and PGE2 concentrations with poly I:C and FSL-1 alone, with a synergistic increase with priming in AECs (Figure 6). The predominant cytokine that was increased was IL-6, a marker of infection associated PTL (97), which has been previously shown to be significantly increased in amnion in women who deliver preterm compared to term (25). Poly I:C has been shown to increase TNF-α, MIP-1α and MIP-β production in fetal membranes by a combination of MyD88 and TRIF dependant and independent mechanisms (98). Although fetal membranes express TLR2, and have the capacity to respond to TLR2 activation, we saw no increase in cytokine or chemokine production in AECs with HKLM. In a study comparing FM explants responses to a panel of TLR agonists, when stimulated with the TLR2 agonist peptidoglycan (PDG), only the cytokine IL-8 was increased, and at lower concentrations when compared with the response to TLR4 and TLR5 agonists (99). PDG, like HKLM, does not require TLR1 or TLR6 to from heterodimers to achieve its effect. We acknowledge that examining primary AECs rather than fetal membrane explants may have limited the response due to the complex bidirectional communications that exist between amnion and the choriodecidua (100). However, by treating AECs, our aim was to mimic the intra-amniotic environment in response to the viral and bacterial components on the cell type that is in the closest proximity to the fetus.

Poly I:C has been shown in several studies to increase mRNA expression of cytokines in myocytes (68–70), consistent with our results. FSL-1 has been shown to be capable of inducing COX-2 mRNA expression and PGF2α production. Although we did not see NF-κB activation FSL-1 in myocytes, we did see a significant increase in p-c-Jun (Supplementary Figures 1, 2). Moreover, we observed a synergistic effect in cytokine and chemokine production with TLR3 viral priming and TLR2/6 agonist stimulation (Figure 7). The implication of this is that IL-8 could serve to chemoattract leukocytes to the myometrium, which is known to occur at the time of term and preterm labour (101). Although there was not a significant increase in PGE2 by the myocytes *in vitro*, leukocytes infiltrating the myometrium *in vivo* would contribute to local prostaglandin synthesis, which in turn could trigger uterine contractility. Additionally, increased IL-6 production by myocytes could lead to the facilitation of uterine contractility since incubation of cultured myocytes with IL-6 increases oxytocin receptor (OTR) mRNA *in vitro* (102). The increase in TNF-α and IL-1β can lead to further activation of NF-κB which in turn leads to a positive feed forward loop further increasing the transcription of pro-labour and pro-inflammatory genes. Additionally, the chemokines macrophage inflammatory protein-1α (MIP-1α), MIP-1β, and RANTES attract immune effector cells which will also lead to amplification of the inflammatory response. Increased local concentrations of RANTES may also induce uterine contractility through induction of basophils to release histamines which are potent uterotonics (103, 104).

Although an augmented effect was seen with the TLR2/6 agonist, we did not see an augmented response to TLR3 priming with TLR2 agonist HKLM or with HKLM treatment alone (Figure 4). This was unexpected, since we had confirmed that HKLM at these concentrations activates both NF-κB and AP-1 (Supplementary Figures 1, 2), and murine studies have shown an increase in sPTB rate with combined TLR2 and 3 agonist treatment (50). It is possible that both agonists are needed to be administered together to achieve synergy, rather than being administered sequentially. It is also plausible that by using different TLR2 agonists, different downstream pathways will be activated, leading to differential effects in TLR2-TLR3 cross-talk. Nevertheless, our data suggests that the lower reproductive tract is more responsive to TLR2/6 activation than to TLR2 alone, and that is responses seen in animal models of systemic infection may not be directly comparable.

Most animal studies of the effect of multi-pathogen induced preterm labour have drawn on models of systemic infection, using intraperitoneal (i.p.) injections. Combined i.p. administration of the murine herpes virus MHV-68 and LPS leads to a 100% PTB rate, in contrast to a 0% PTB rate with MHV-68 alone, and 29% LPS alone (48). Similarly, i.p. of the TLR2 ligand peptidoglycan PGN and poly I:C leads to a 100% PTL, compared to 15% and 22% in Poly I:C or PGN alone, respectively (50). We compared the production of cytokines, chemokines and PGE2 in response to either TLR3, TLR2, or TLR2/6 agonists alone, with the response to TLR2 or TLR2/6 agonists following TLR3 priming in PBMCs as a model of systemic infection and inflammation, and in placental explants as a model of haematogenous spread of multi-pathogen infection. We selected PBMCs since lymphocytes and monocytes are key immune cells for regulating the response to viruses and bacteria. We used PBMC conditioned media to culture placental explants in order to reflect the high circulating volume through the placenta and to assess the effect of systemic inflammation on placental tissue.

PBMCs exposed to TLR3 viral priming showed augmented production of IL-1β, IL-6, IL-8, TNF-α, and PGE2 when incubated with both the TLR2 and TLR2/6 agonists (Figure 10). This is consistent with the reported synergy between the Pam_3_CSK_4_ TLR2 agonist and poly I:C seen in murine dendritic cells in pro-inflammatory cytokine production, NK cell derived IFN-γ production and increased T cell proliferation (105). However, in contrast, placental explants did not show an augmented pro-inflammatory cytokine response to dual treatment. To the contrary, a significant increase in IL-10 concentrations were seen with TLR3 agonist priming prior to stimulation with TLR2 and TLR2/6 agonists (Figures 11, 12). This implies that the placenta can form a barrier to local multi-pathogen exposure. However, placental explants did elicit a pro-inflammatory response following single agonist (TLR2, TLR2/6, and TLR3) exposure. This data is consistent with that of several studies exploring the functional role of TLRs in human trophoblast and placental explants (106–109). However, we also report on an increase in the production of PGE2 following the exposure to each of the agonists, which could act locally on fetal membranes and myometrium leading to membrane rupture and uterine contractility. Furthermore, when placental explants were grown in PBMC conditioned media from priming experiments, an increase in IL-8 and IL-6 was seen with TLR3/TLR2 PBMC conditioned media (Figure 13). The clinical implication of this finding could relate to the well-recognised risk of neonatal brain injury that is associated with the presence of systemic maternal inflammation (110, 111).

Finally, we examined the effect of poly I:C on TLR3, TLR2, and TLR6 expression (Figure 14). Poly I:C induced the expression of both TLR3 and TLR2, except in placental explants, but no effect was seen on TLR6 expression. We conclude that TLR2 is likely to be the rate limiting step in TLR2/TLR6 dimer formation necessary for the synergism seen with poly I:C and FSL-1 incubation. We hypothesise that increasing TLR2 expression results in increased dimerization with TLR6. In contrast, the TLR2 agonist HKLM does not require heterodimerization exert activity, and our data would suggest that the degree of receptor expression of TLR2 does not influence its efficacy. To lend support to the increased synergy seen with the TLR2/6 agonist compared to the more pure TLR2 agonist, mice treated with lipoteichoic acid (TLR2/6 agonist) show higher rates of preterm birth compared with those treated with peptidoglycan (PGN) which requires only TLR2 dimerization to exert its effect (39). In addition, mice treated with Pam2Cys (reliant on TLR2/6 dimerization) showed higher preterm birth rates and excessive proinflammatory cytokine production compared with Pam3Cys (reliant on TLR2/1) (112). Future work on the effect of viral priming with TLR3 prior to TLR2 agonist treatment on the formation of TLR2/1 and TLR2/6 heterodimers would provide further mechanistic insight into the role of TLRs in multi-pathogen induced preterm labour.

**Figure 15:**
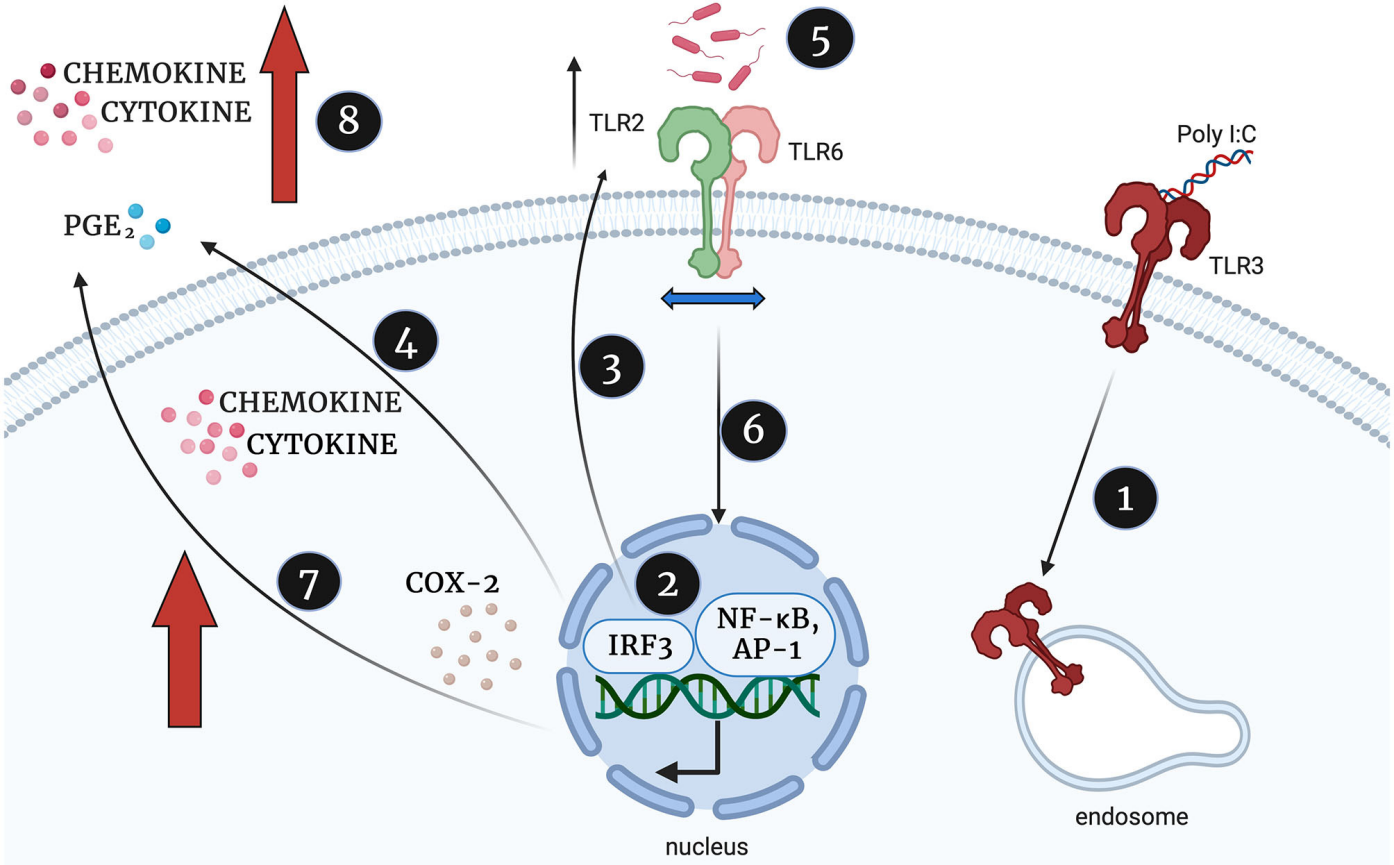
Proposed mechanism of synergistic increase in cytokine, chemokine and PGE2 production in TLR3 primed and TLR2/6 treated cells, created with BioRender.com. Poly I:C activates TLR3 (1) which leads to TLR3 mediated activation of NF-κB, AP-1 and p-IRF3 (2). NF-κB, AP-1 and IRF-3 increases TLR2 expression (3) and stimulates the production of cytokines, COX-2 and PGE2, (4). The TLR2/6 agonist shows increased potency due to increased availability of TLR2 to dimerise with TLR6 (5), TLR2/6 leads to increased NF-κB activation (6) and augmented production of cytokines, COX-2 and PGE2 production (7). This leads to an uncontrolled positive feed forward loop and triggering uterine contractility, fetal membrane rupture and cervical dilation and ultimately a multi-pathogen induced preterm labour.

In summary, and illustrated in Figure 15, we propose that viral stimulation of TLR3 leads to activation of NF-κB, AP-1, and p-IRF3, increased TLR2 expression and increased production of cytokines, COX-2 and PGE2. We hypothesise that this initial TLR3 priming increases the availability of TLR2 to dimerise with TLR6 leaving target cells more susceptible to bacterial sensing via TLR2/6. As a result, further activation of NF-κB, AP-1 and p-IRF3, leads to augmented production of cytokines, COX-2 and PGE2 sufficient to trigger uterine contractility, fetal membrane rupture and cervical dilation and ultimately a multi-pathogen induced preterm labour.

## Conclusions

Although our study confirms that the placenta has the capacity to mount a pro-inflammatory response to single pathogen exposure, multi-pathogen exposure does not cause an augmented pro-inflammatory effect but does cause an increase in the anti-inflammatory cytokine IL-10. We hypothesise that this response is needed at the maternal-fetal interface to form a protective barrier for the fetus to allow time for the mother to recover from her systemic illness, whilst keeping the fetus *in utero*. This response is in contrast to the heightened pro-inflammatory and pro-labour mediator responses of VECs and AECs seen in our model of ascending multi-pathogen infection. This response is in keeping with clinical cases of chorioamnionitis secondary to ascending infection, where often the inflammatory response drives labour and delivery to expel the fetus to protect the mother. We conclude that viruses lead to modulation of TLR mediated cellular signalling responses that could increase susceptibility to bacterial induced preterm birth, and that this is likely to be more pronounced in cases of ascending rather than systemic infection. Future, *in vivo*, experimental medicine and clinical studies should explore the potential role of local or systemic viral infection in the aetiology of sPTL.

## Data Availability Statement

The raw data supporting the conclusions of this article will be made available by the authors, without undue reservation.

## Ethics Statement

The studies involving human participants were reviewed and approved by Hammersmith, Queen Charlotte's & Chelsea Hospitals Research Ethics Committee (Ref 2002/628) and Riverside Research Ethics Committee (Ref 3358) and South East London Ethics Committee (Ref 10/H0805/54). The patients/participants provided their written informed consent to participate in this study.

## Author Contributions

ZR, LS, DM, PB, and MS were responsible for the conception and design of the study. ZR, RR, CR, EA, YL, SK, and LS conducted experiments and data analysis. All figures and tables were created by ZR. ZR drafted the manuscript and all authors critically reviewed it. All authors contributed to the article and approved the submitted version.

## Conflict of Interest

The authors declare that the research was conducted in the absence of any commercial or financial relationships that could be construed as a potential conflict of interest.

## Supplementary Material

Supplementary Figure 1 Effect of HKLM (TLR2 agonist) and FSL-1 (TLR2/6 agonist) phospho-65 in VECs, AECs, myocytes and PBMCs. Cells were treated with TLR2 (HKLM) orTLR2/6 (FSL-1) agonists and protein was extracted to quantify the expression level of NF-κB activation via Western immunoblotting. VECs, AECs, and myocytes were treated with 10^5^ cells/ml and 10^8^ cells/ml of HKLM in PBMCs. VECs and AECs were treated with 0.01 μg/ml of FSL-1 and myocytes, PBMCs were treated with 0.1 μg/ml. Stimulation was performed for over 24 h and protein was extracted and quantified using immunoblotting for phosphorylated p-65. P-p65 increased significantly at 4, 6, 12, and 24 h with HKLM **(A)** and 30 min, and 1 h with FSL-1 in VECs **(B)**. In AECs, p-p65 increased significantly at 30 min, 1 and 4 h with HKLM **(C)** and 30 min and 1 h with FSL-1 **(D)**. Myocytes only showed a significant increase with HKLM at 1 h **(E)** while a non-significant increase was seen at 4 h with FSL-1 **(F)**. PBMCs also showed a significant increase at 24 h with HKLM **(G)** and a trend of increase with FSL-1 **(H)**. For statistical analysis One-way ANOVA was used for normally distributed data and Kruskal-Wallis was used for data not normally distributed with Dunnett's multiple comparison test *n* = 3–6.

Supplementary Figure 2 Effect of HKLM (TLR2 agonist) and FSL-1 (TLR2/6 agonist) on p-c-Jun in VECs, AECs, myocytes, and PBMCs. Cells and placental explants were treated with TLR2 (HKLM) orTLR2/6 (FSL-1) agonists and protein was extracted to activation of AP-1 via Western immunoblotting. VECs, AECs, and myocytes were treated with 10^5^ cells/ml and 10^8^ cells/ml of HKLM in PBMCs and placental explants. VECs and AECs were treated with 0.01 μg/ml of FSL-1 and myocytes, PBMCs and placental explants were treated with 0.1 μg/ml. Stimulation was performed for over 24 h and protein was extracted and quantified using Western immunoblotting for p-c-Jun. P-c-Jun increased significantly at 30 min, 1 h, and 24 h with HKLM **(A)** and 30 min and 1 h with FSL-1 in VECs **(B)**. In AECs, p-c-Jun increased significantly at 30 min and 1 h with HKLM **(C)** and 30 min, 1, 4, and 6 h with FSL-1 **(D)**. Myocytes showed a significant increase with HKLM at 1 and 4 h **(E)** while a significant increase was seen at 4 h with FSL-1 **(F)**. For statistical analysis One-way ANOVA was used for normally distributed data and Kruskal-Wallis was used for data not normally distributed with Dunnett's multiple comparison test *n* = 3–6.

Supplementary Figure 3 Original western blots that we used in the cut blots from Figure 8. Activation of NF-κB and AP-1 in placental explants. The blots were taken from a priming experiment of placental explants with 25 μg/ml of poly I:C for 4 h prior to 4 h stimulation with 10^8^ cell/ml of TLR2 agonist HKLM, 0.1 μg/ml the TLR4 agonist LPS or 0.1 μg/ml of the TLR2/6 agonist FSL-1 with their respective non-stimulated controls. The red histograms in both graphs reflect the summary results shown in Figure 8. The immunoblot results were cut and presented in Figure 8.
