# Supplementary figures and images for: Differential Response of Gestational Tissues to TLR3 Viral Priming Prior to Exposure to Bacterial TLR2 and TLR2/6 Agonists
Source: Front Immunol. 2020 Aug 25;11:1899. doi: 10.3389/fimmu.2020.01899 (PMC7477080; doi:10.3389/fimmu.2020.01899)

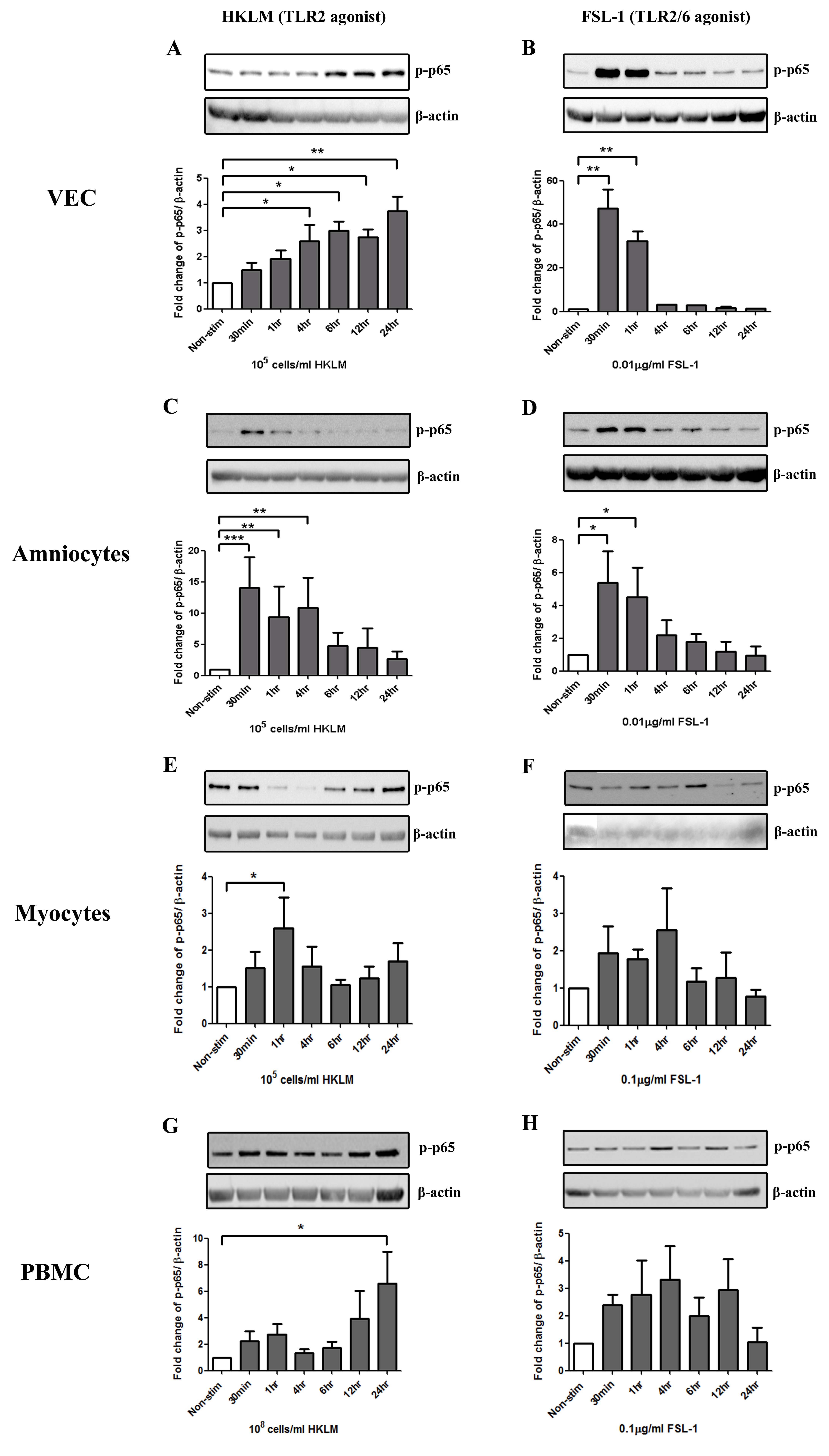

Supplement: Supplementary Figure 1 — Effect of HKLM (TLR2 agonist) and FSL-1 (TLR2/6 agonist) phospho-65 in VECs, AECs, myocytes and PBMCs. Cells were treated with TLR2 (HKLM) orTLR2/6 (FSL-1) agonists and protein was extracted to quantify the expression level of NF-κB activation via Western immunoblotting. VECs, AECs, and myocytes were treated with 105 cells/ml and 108 cells/ml of HKLM in PBMCs. VECs and AECs were treated with 0.01 μg/ml of FSL-1 and myocytes, PBMCs were treated with 0.1 μg/ml. Stimulation was performed for over 24 h and protein was extracted and quantified using immunoblotting for phosphorylated p-65. P-p65 increased significantly at 4, 6, 12, and 24 h with HKLM (A) and 30 min, and 1 h with FSL-1 in VECs (B). In AECs, p-p65 increased significantly at 30 min, 1 and 4 h with HKLM (C) and 30 min and 1 h with FSL-1 (D). Myocytes only showed a significant increase with HKLM at 1 h (E) while a non-significant increase was seen at 4 h with FSL-1 (F). PBMCs also showed a significant increase at 24 h with HKLM (G) and a trend of increase with FSL-1 (H). For statistical analysis One-way ANOVA was used for normally distributed data and Kruskal-Wallis was used for data not normally distributed with Dunnett's multiple comparison test n = 3–6. [file Image_1.JPEG]

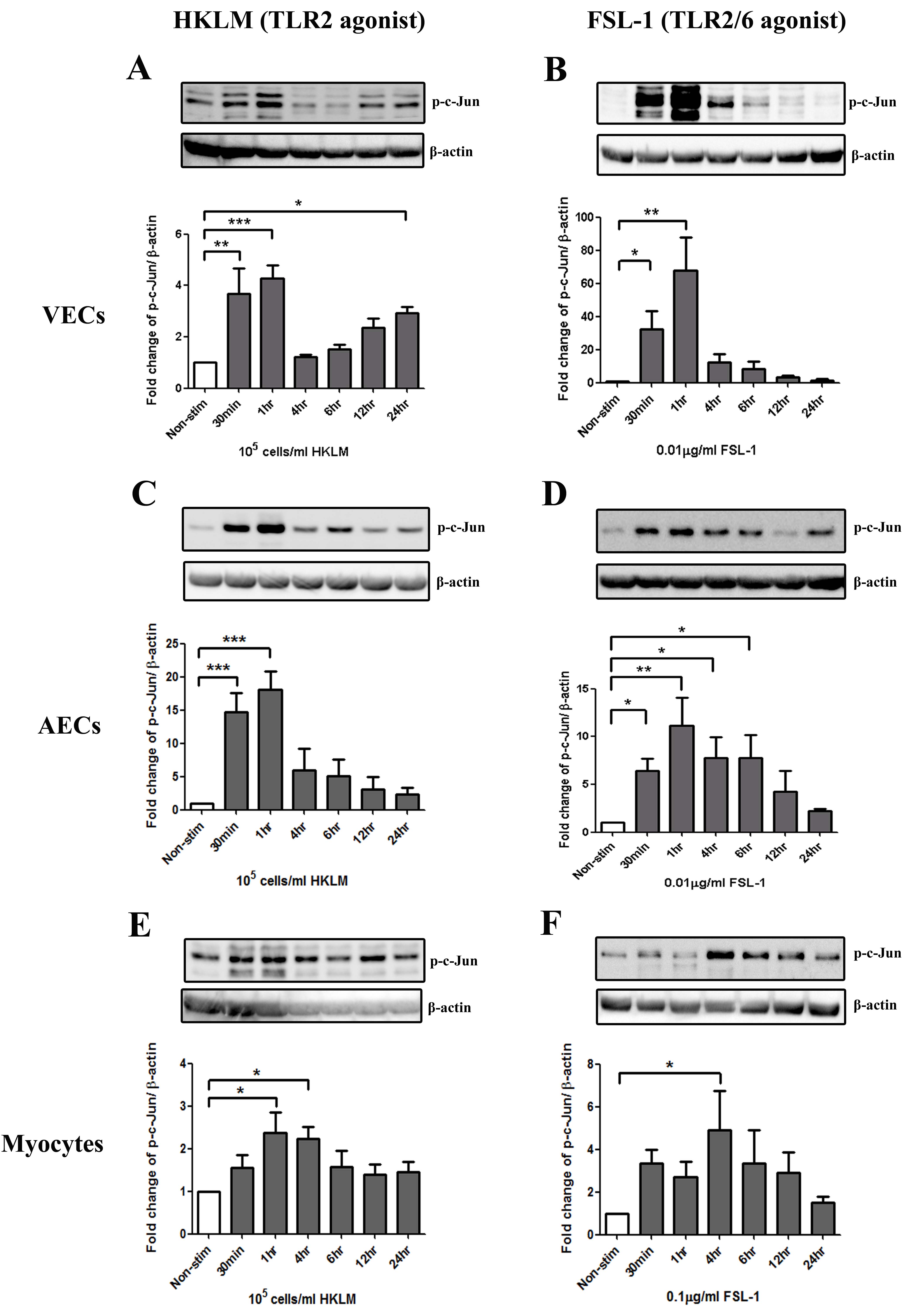

Supplement: Supplementary Figure 2 — Effect of HKLM (TLR2 agonist) and FSL-1 (TLR2/6 agonist) on p-c-Jun in VECs, AECs, myocytes, and PBMCs. Cells and placental explants were treated with TLR2 (HKLM) orTLR2/6 (FSL-1) agonists and protein was extracted to activation of AP-1 via Western immunoblotting. VECs, AECs, and myocytes were treated with 105 cells/ml and 108 cells/ml of HKLM in PBMCs and placental explants. VECs and AECs were treated with 0.01 μg/ml of FSL-1 and myocytes, PBMCs and placental explants were treated with 0.1 μg/ml. Stimulation was performed for over 24 h and protein was extracted and quantified using Western immunoblotting for p-c-Jun. P-c-Jun increased significantly at 30 min, 1 h, and 24 h with HKLM (A) and 30 min and 1 h with FSL-1 in VECs (B). In AECs, p-c-Jun increased significantly at 30 min and 1 h with HKLM (C) and 30 min, 1, 4, and 6 h with FSL-1 (D). Myocytes showed a significant increase with HKLM at 1 and 4 h (E) while a significant increase was seen at 4 h with FSL-1 (F). For statistical analysis One-way ANOVA was used for normally distributed data and Kruskal-Wallis was used for data not normally distributed with Dunnett's multiple comparison test n = 3–6. [file Image_2.JPEG]

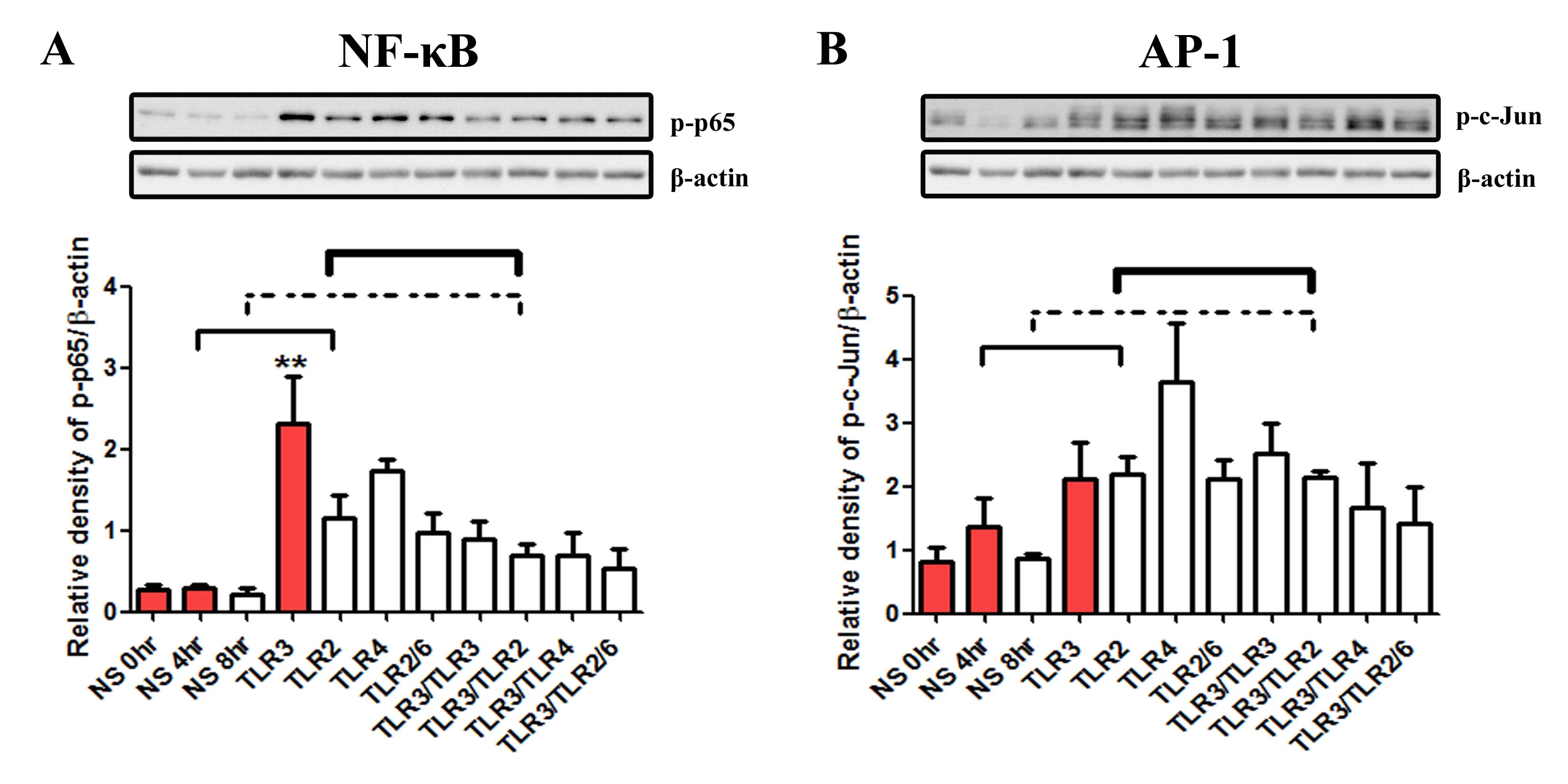

Supplement: Supplementary Figure 3 — Original western blots that we used in the cut blots from Figure 8. Activation of NF-κB and AP-1 in placental explants. The blots were taken from a priming experiment of placental explants with 25 μg/ml of poly I:C for 4 h prior to 4 h stimulation with 108 cell/ml of TLR2 agonist HKLM, 0.1 μg/ml the TLR4 agonist LPS or 0.1 μg/ml of the TLR2/6 agonist FSL-1 with their respective non-stimulated controls. The red histograms in both graphs reflect the summary results shown in Figure 8. The immunoblot results were cut and presented in Figure 8. [file Image_3.JPEG]
